# The role of intracellular trafficking of CdSe/ZnS QDs on their consequent toxicity profile

**DOI:** 10.1186/s12951-017-0279-0

**Published:** 2017-06-15

**Authors:** Bella B. Manshian, Thomas F. Martens, Karsten Kantner, Kevin Braeckmans, Stefaan C. De Smedt, Jo Demeester, Gareth J. S. Jenkins, Wolfgang J. Parak, Beatriz Pelaz, Shareen H. Doak, Uwe Himmelreich, Stefaan J. Soenen

**Affiliations:** 1 Biomedical NMR Unit/MoSAIC, KU Leuven Campus Gasthuisberg, Herestraat 49, 3000 Louvain, Belgium; 2 Institute of Life Science, Swansea University Medical School, Singleton Park, Swansea, SA2 8PP UK; 3 Faculty of Pharmaceutical Sciences, Ghent University, Harelbekestraat 72, 9000 Ghent, Belgium; 4 Center of Nano- and Biophotonics, Ghent University, Harelbekestraat 72, 9000 Ghent, Belgium; 5 Philipps University of Marburg, Renthof 7, 35032 Marburg, Germany; 6 CICBiomagune, San Sebastian, Spain

**Keywords:** Quantum dot NPs, Intracellular localization, Endosomal uptake, Gene alterations, Nanotoxicity

## Abstract

**Background:**

Nanoparticle interactions with cellular membranes and the kinetics of their transport and localization are important determinants of their functionality and their biological consequences. Understanding these phenomena is fundamental for the translation of such NPs from in vitro to in vivo systems for bioimaging and medical applications. Two CdSe/ZnS quantum dots (QD) with differing surface functionality (NH_2_ or COOH moieties) were used here for investigating the intracellular uptake and transport kinetics of these QDs.

**Results:**

In water, the COOH- and NH_2_-QDs were negatively and positively charged, respectively, while in serum-containing medium the NH_2_-QDs were agglomerated, whereas the COOH-QDs remained dispersed. Though intracellular levels of NH_2_- and COOH-QDs were very similar after 24 h exposure, COOH-QDs appeared to be continuously internalised and transported by endosomes and lysosomes, while NH_2_-QDs mainly remained in the lysosomes. The results of (intra)cellular QD trafficking were correlated to their toxicity profiles investigating levels of reactive oxygen species (ROS), mitochondrial ROS, autophagy, changes to cellular morphology and alterations in genes involved in cellular stress, toxicity and cytoskeletal integrity. The continuous flux of COOH-QDs perhaps explains their higher toxicity compared to the NH_2_-QDs, mainly resulting in mitochondrial ROS and cytoskeletal remodelling which are phenomena that occur early during cellular exposure.

**Conclusions:**

Together, these data reveal that although cellular QD levels were similar after 24 h, differences in the nature and extent of their cellular trafficking resulted in differences in consequent gene alterations and toxicological effects.

**Electronic supplementary material:**

The online version of this article (doi:10.1186/s12951-017-0279-0) contains supplementary material, which is available to authorized users.

## Background

The scope of the use of nanomaterials (NMs) not only for technological, but also in biomedical and clinical applications is still increasing, where mainly imaging purposes and more recently therapeutic purposes are being explored to greater depth. This is driven by the high number of unique physical and chemical properties that many materials possess when downsized to the nanoscale. One such type of NM are quantum dots (QDs), which are small colloidal semiconductor nanoparticles (NPs) that possess remarkable photophysical properties, including high photostability and brightness, along with very narrow and size-tunable emission spectra [1, 2]. These properties have enabled the real-time tracking of surface-located receptors in live cells over longer time periods [3, 4], as well as intracellular tracking of single molecules and protein [5–7]. QDs also have potential as probes for in vivo fluorescence imaging [8]. They are being explored as therapeutic agents [9], such as in photodynamic therapy where the QDs could be used to eradicate cancer cells [10]. Despite alternative materials, the predominantly used QDs are based on II/VI group semiconductor materials, and thus typically comprise Cd. Given their chemical composition and the presence of highly toxic elements such as Cd^2+^ [11, 12], the use of QDs in live cells, tissues, and clinical applications has remained limited. Despite various strategies being explored to reduce their toxicity (e.g. Cd^2+^-free QDs, dual polymer-silica coated QDs), their practical use in biomedical applications remains moderate. This is in part due to the absence of sufficient information about the precise mechanisms and kinetics involved in the interaction of QDs with biological entities. Some recent studies have tackled this topic [13–15] yet more research is required to understand the effects of specific physico-chemical differences in NPs on their toxicity profiles [16]. Additionally, one inherent issue with the field of nanosafety research is the near endless number of potential interactions of NPs with biological components, of which only a selected few can be examined in every single study for a selected in vitro or in vivo model [17]. As most studies will focus on key mechanisms, such as the induction of reactive oxygen species (ROS) or gross cell viability studies, more subtle effects are often overlooked and differences between the various in vitro and in vivo models used can drastically alter the outcome of any study [18, 19]. As such, several key questions regarding the potential toxic effects of QDs remain thus far not fully answered.

In the present work, two different types of QDs (bearing negative and positive surface charge) are being used to examine cyto- and genotoxic effects on cultured human cells. Continuing on the results obtained in a previous work with the same QDs [20], further investigations are performed here to evaluate the kinetics of their cellular uptake, intracellular localization, and the alterations they induce to the cellular homeostasis in an effort to attaining a better understanding of the observed differences in their toxicity profiles. Intracellular cadmium levels are quantified and correlated to changes in cellular homeostasis. One major aim of this study is therefore to link the differences in physicochemical parameters with the kinetics of cellular processing and their toxicity levels. A second aim is to further elucidate upon the mechanisms by which the different QDs exert their toxicity. For this purpose, the effect of the intracellular environment on QD functionality and chemical stability are investigated. Additionally, detailed gene expression studies are performed and activation of important cytoskeletal regulator and stress and toxicity signalling pathways are examined. Finally, all results are combined and analysed together, in order to evaluate whether the differences in physicochemical properties of the QDs are linked to their respective uptake kinetics and levels, and whether their intracellular processing also influences QD behaviour and their mechanism of toxicity.

Therefore, this study is a more comprehensive investigation and exploration of the processes responsible for the differences in the cellular and NP interactions that was previously published using the same QDs [20].

## Results

### Properties of the QDs

The COOH- and NH_2_-CdSe/ZnS QDs were purchased from different vendors but both NPs had the same core and surface coating. The diameter of the inorganic part (i.e. the CdSe core and the ZnS shell, excluding the organic surface coating) of the QDs was determined as 4.6 ± 0.5 nm for the carboxyl, COOH-QDs (QD−) and 6.9 ± 0.9 nm for the amine, NH_2_-QDs (QD+) (for details please see Additional file 1: Figure S1). The emission spectra were different for the different QDs where COOH-QDs had their first excitation peak at 585 and 664 nm for the NH_2_-QDs. At the same QD concentration and at 450 nm excitation the COOH-QDs were much brighter than the NH_2_-QDs. In water, the COOH- and NH_2_-QDs were negatively and positively charged, respectively. In serum-containing medium the NH_2_-QDs were agglomerated (as indicated by the largely increased hydrodynamic diameter), whereas the COOH-QDs remained dispersed (for more QD characterisation information please see the supporting information, a summary is given in Additional file 1: Table S7).

### Cellular uptake by confocal microscopy

**Fig. 1:**
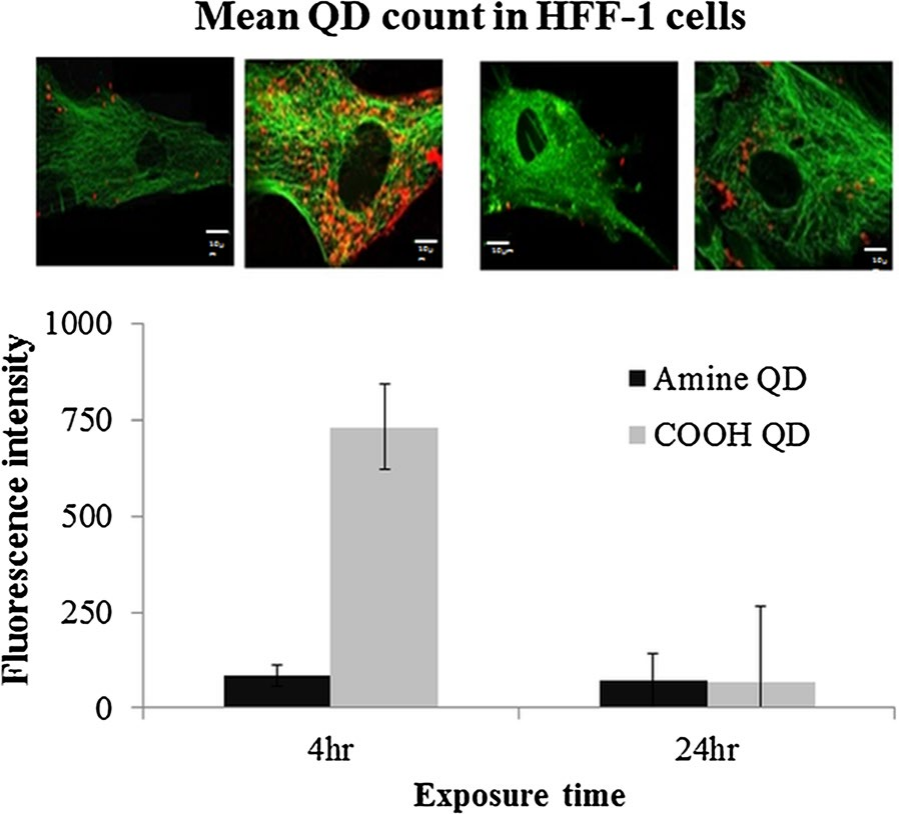
Graph representing semi-quantitative results of fluorescence intensity of QDs detected in HFF-1 cells following 4 and 24 h exposure. Data are expressed as mean ± standard error of the mean (SEM, *n* = 10). The inserts are representative confocal microscopy images of tubulin (*green*) stained cells exposed to the respective QDs (*red*) at 7.5 nM QD concentration. *Scale bars* correspond to 10 μm

Quantum dots internalisation by HFF-1 cells following 4 and 24 h exposure was examined by confocal microscopy of tubulin stained cells. QDs were confirmed to be in the cells by 3D imaging. Both, NH_2_- and COOH-QDs were readily taken up by the cells, as observed from the images (Fig. 1). However, upon semi-quantification of cellular QD levels, clear differences in fluorescence levels were observed after 4 h, where COOH-QDs resulted in higher cellular fluorescence. After 24 h, fluorescence had however dropped significantly, reaching the same level as the NH_2_-QDs. The NH_2_-QDs did not show any significant differences between 4 and 24 h exposure and appeared to rapidly reach maximum intracellular fluorescence levels.

### Alterations to QD properties with varying pH conditions

The effects of altering pH levels on the QD properties were tested here by dissolving the NPs in citrate containing PBS the pH of which was adjusted to 4.5, 5.5, and 7.4 (please see Additional file 1: Figure S11). Our results showed that the fluorescence of the COOH-QDs is indeed quenched after 48 h at all pH levels and was most prominent at the lower pH levels, but the overall effects were strong in all conditions (Additional file 1: Figure S11A). In contrast, NH_2_-QDs showed no degradation effects of fluorescence following incubation with the three solutions for up to 4 days (Additional file 1: Figure S11B).

### Determination of QD properties upon cellular internalization

To evaluate whether the semiconductor part of the QDs dissolved in the cellular environment, the Measure-IT (Invitrogen Ltd. UK) commercially available kit was used to assess free cadmium ion content in HFF-1 cells treated with COOH- or NH_2_-QDs for 24 h. In order to assess the effect of the low endosomal pH on the QDs, non-proliferating HFF-1 cells were used, as highly proliferative cells would have complicated this analysis by the continuous dilution of both intracellular QDs and intracellular free ions [21]. Data (Additional file 1: Figure S11) revealed significant increases in cellular free Cd^2+^ levels in COOH- and NH_2_-QD treated cells at all the tested time points, starting at day 2, for COOH-QDs and starting from day 3 for NH_2_-QDs.

### Evaluation of cellular QD trafficking

#### Confocal microscopy

**Fig. 2:**
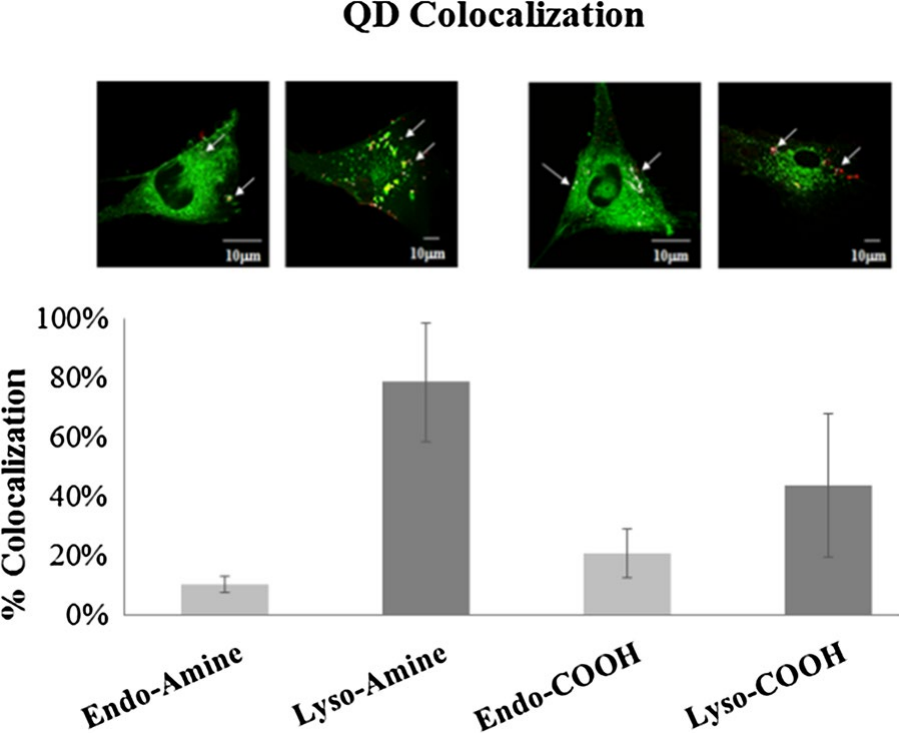
Graph representing results of colocalization analysis using the JACoP plugin from ImageJ using Manders’ correlation coefficient. The thresholded Mander’s M values corresponding to the fraction of QDs in the lysosomes (“Lyso”) or endosomes (“Endo”) following 24 h exposure are shown. Results are presented as the mean ± SEM (*n* = 10). Representative confocal images of colocalized (*white points*) QDs with endosomes or lysosomes are presented above *each bar*. Examples of colocalized points are indicated with *white arrows*. *Scale bars* correspond to 10 μm

Cellular interaction of the two QDs was studied using confocal microscopy based analysis of cells expressing green fluorescent protein (GFP)-tagged Lamp1 (lysosomal marker) or EEA1 (marker for early endosomes). Colocalization between either QD and Lamp1 or EEA1 was determined from the acquired images using the ImageJ analysis tool. Lysosomes or endosomes were considered as colocalized with the QDs when their respective intensities were higher than the threshold of their individual channels and if their ratio of intensity was more than the ratio setting value [22]. Figure 2 reveals that after 24 h incubation, there is a clear difference between the two types of QDs, where NH_2_-QDs result in much higher levels of QDs colocalizing with lysosomes. In contrast, COOH-QDs result in much higher levels of QDs colocalizing with early endosomes.

#### Fluorescence single particle tracking

**Fig. 3:**
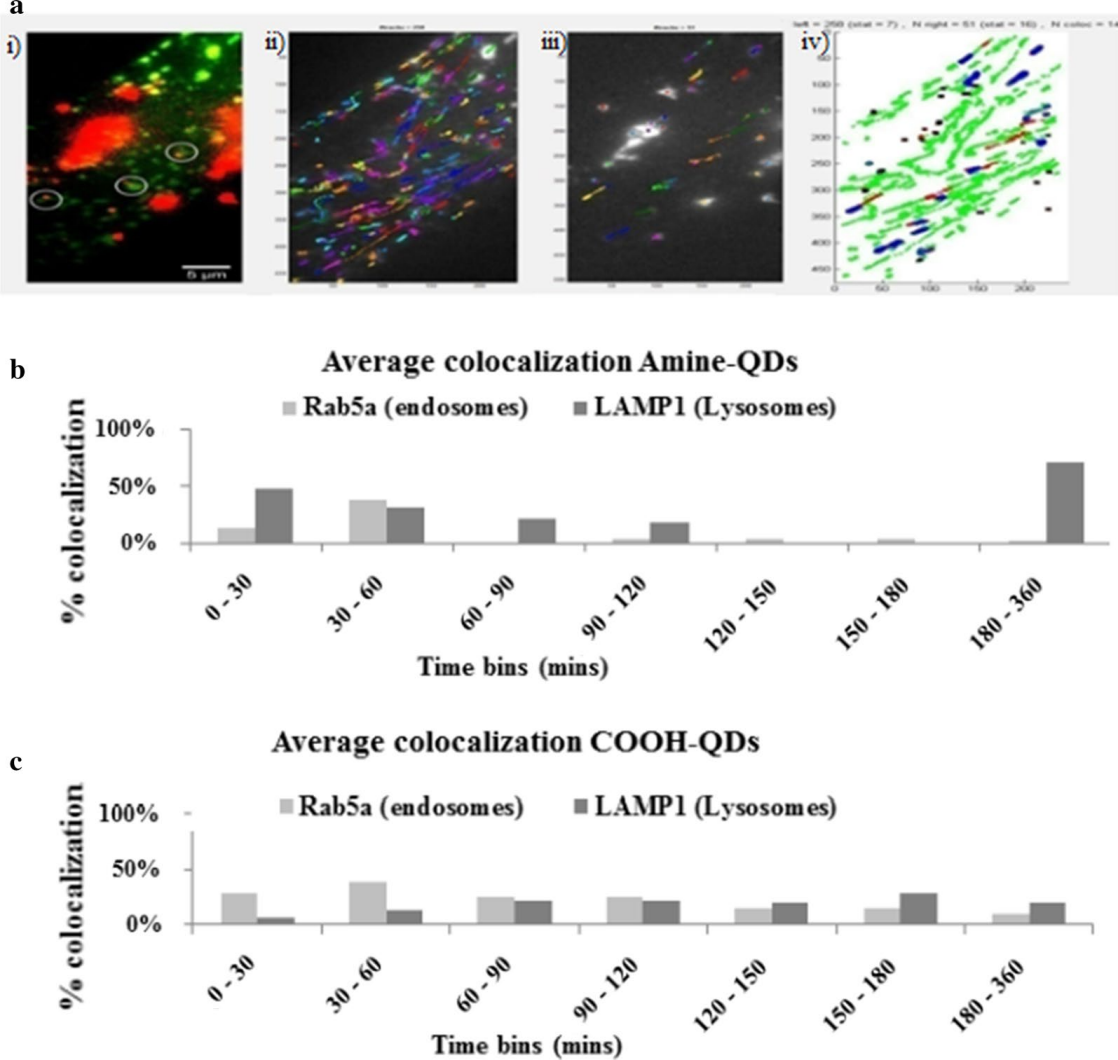
Plots from the intracellular trafficking profile of QDs in HFF-1 cells using early endosomes-GFP, and lysosomes-GFP. **a** Images showing an example of the (*i*) overlay of NH_2_-QDs with the lysosomal marker, (*ii*) the tracks for the *green* lysosomal channel, (*iii*) tracks for the QD channel, and (*iv*) colocalization of *green* (lysosomal) and *red* (QD) tracks. The* scale bar* corresponds to 5 μm. **b**, **c** Graphs represent trajectory-based dynamic colocalization of fluorescent NH_2_-QDs with the endosomal marker (Rab5a) and lysosomal marker (LAMP1) that was calculated and *plotted* as a function of time.* Each dot* corresponds to 1 min movie recording that was taken in different cells at that specific time point

In this analysis, the two QDs showed different profiles of uptake and localization in the intracellular environment at the different time points (Fig. 3). NH_2_-QDs were taken up by the Rab5a-positive early endosomes with endosomal colocalization increasing with time until 1 h post exposure. They then appeared to be immediately transported into the LAMP1-containing organelles, being mainly lysosomes (Fig. 3a), where the majority of these QDs remained until 6 h post exposure. The QDs that were not found to be colocalized with Rab5a or LAMP1 (at 120–180 min time points) were likely present in intermediary organelles such as late endosomes [23]. After 3–6 h, the majority of the detected QDs were found in the lysosomal compartment. The COOH-QDs displayed a completely different profile, where up to 6 h only a low number of QDs were present in the lysosomal compartment (Fig. 3b).

#### Exocytosis investigation with ICP-MS

**Fig. 4:**
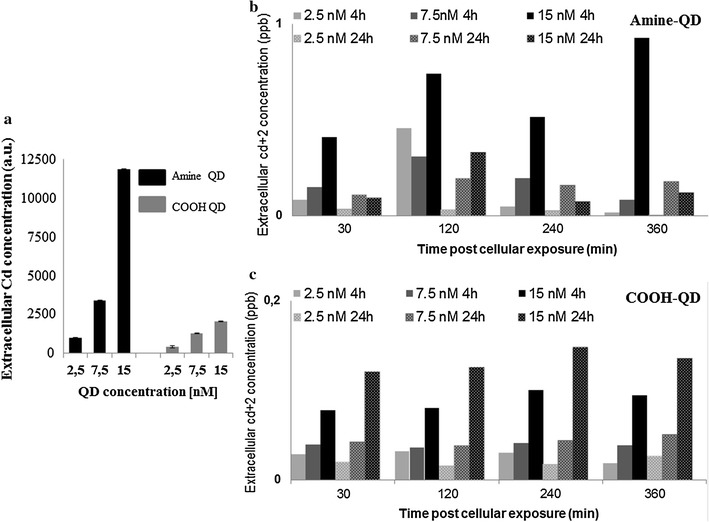
**a** Graph representing the amount of elemental Cd remaining in the cell culture medium for each exposure concentration following 4 h incubation. **b**, **c** Figures showing the* number* of elemental Cd, relative to the control, detected in the cell culture media at each concentration after 4 h (*solid filled bars*) and 24 h (*dotted bars*) incubation, which was followed by immediate washing of the cells. Samples were collected and measured with ICP-MS at the different time points. Please note the difference in the* y*-scale between graphs **b** and **c**

The results of this analysis (Fig. 4) revealed clear differences in the cellular release of Cd^2+^ ions by HFF-1 cells, depending on the type of QD. Figure 4a shows that after 4 h the cell culture media contained sixfold higher amounts of Cd^+2^ ions following exposure to the NH_2_-QDs as compared to the COOH-QDs. Higher number of exocytosed NH_2_-QDs as compared to the COOH-QDs. COOH-QDs demonstrated dose and time dependent increase in the level of QDs released into the cell culture media (Fig. 4c). On the other hand, exocytosis of NH_2_-QDs did not have a time dependent pattern however there was a dose dependency in the 4 h treatments up to 6 h post removal of the NPs from the culture media. This pattern has disappeared in the 24 h experiments (Fig. 4b).

### Evaluation of QD induced cellular stress

**Fig. 5:**
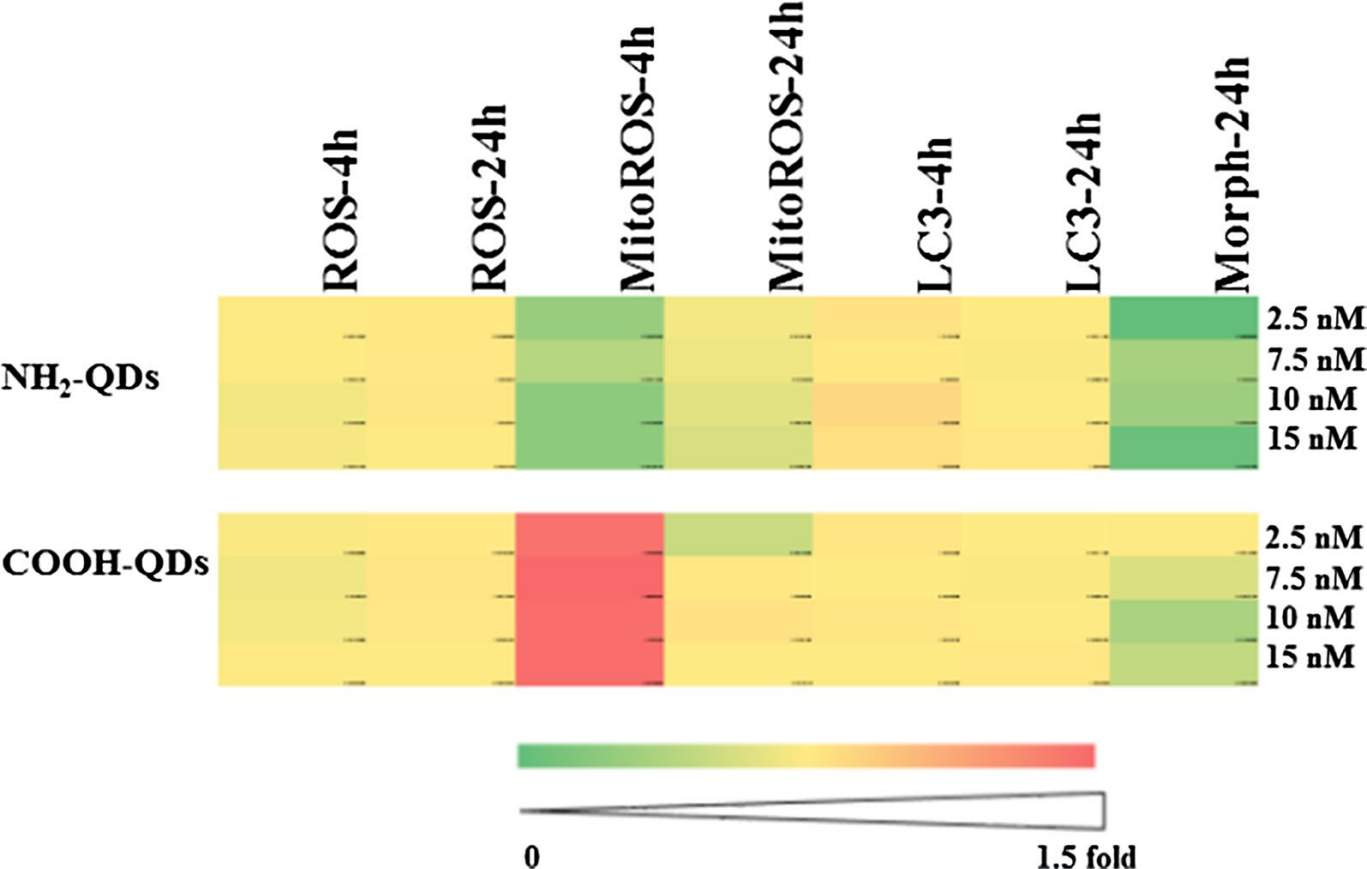
A heat map of the level of toxicity detected with the different toxicity screening assays upon exposure of the cells to the NH_2_-QDs or COOH-QDs at 2.5, 7.5, 10, and 15 nM concentrations

Next, the toxic effects of the QDs were evaluated following 4 and 24 h exposure, using an already validated high-content imaging approach [14], where a few parameters were selected at sub-cytotoxic concentrations, which were defined in another work [24]. These were the levels of reactive oxygen species (ROS), mitochondrial ROS, induction of autophagy, and alterations to cell morphology. A heat-map is used here to compare toxicity profiles between both types of QDs. For this analysis, the control values were all normalised to 100%. The data show no major effect of the NH_2_-QDs at any of the parameters tested (Fig. 5, for more detailed results and images see Additional file 1: Figures S12–14). The COOH-QDs however resulted in induction of mitochondrial ROS (Additional file 1: Figure S14) and reduction in cell area after 24 h exposure (Additional file 1: Figure S13). Neither of the two QD types resulted in a significant effect on cellular autophagy. Neither of the QDs tested here resulted in significant induction of ROS. However, one should keep in mind that there are different types of ROS that can be generated by various processes in different cellular compartments. Here, the mitochondrial-specific probe did indicate induction of mitochondrial ROS, even at the lowest concentration of COOH-QDs. Interestingly, increasing the QD concentration did not correlate with higher levels of mitochondrial ROS, as under the conditions used, a near-constant high level of mitochondrial ROS was observed, when cells were exposed to the COOH-QDs.

### Gene expression studies

**Fig. 6:**
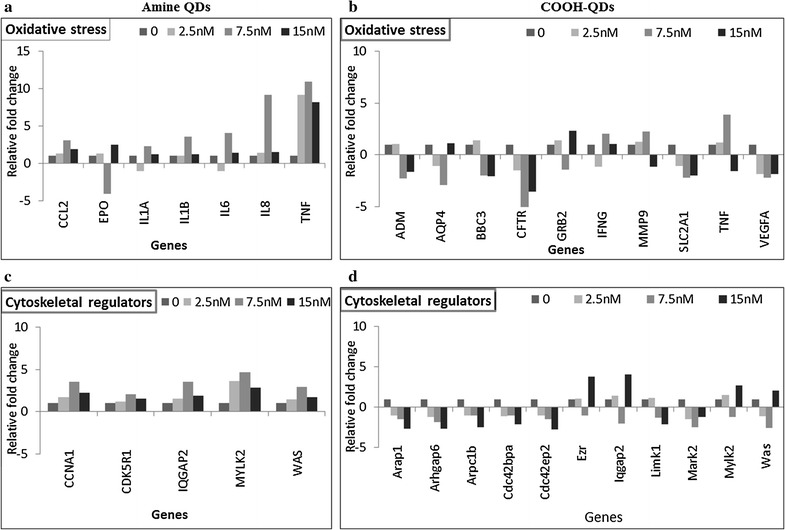
Graphs showing relative gene expression changes in HFF-1 cells exposed to either COOH- or NH_2_-QDs at 0, 2.5, 7.5 or 15 nM concentrations for 24 h. Concentrations were selected where no significant toxicity were detected, along with the negative control. All genes tested are genes involved in the human oxidative stress pathway (**a**, **b**) and the human cytoskeletal regulator gene pathway (**c**, **d**). Only those genes are shown in which for at least one of the tested concentrations a more than twofold change was detected. Data are expressed as the fold-change in mean gene expression values, normalized to the values obtained in untreated control cells

In the arrays investigating genes involved in cellular stress and toxicity different sets of genes were found to be up- or down-regulated following 24 h exposure to NH_2_- or COOH-QDs. NH_2_-QD exposure resulted in an increase in CCL2, IL1A, IL1B, IL6, IL8, and TNFα genes (Fig. 6a). In contrast, exposure to the higher concentrations (7.5 and 15 nM) of COOH-QDs resulted in the downregulation of several genes mainly involved in the hypoxic processes (Fig. 6b). The most significant of these genes included CFTR, AQP4, and ADM, all of which demonstrated no notable changes from exposure to the NH_2_-QDs. Significant changes were defined as at least twofold changes as compared to untreated control levels. Another important difference between the two QDs was the significant downregulation of VEGFA recorded with the COOH-QDs which was absent from exposure to the NH_2_-QDs. On the other hand, like its counterpart, COOH-QD resulted in the upregulation of TNF gene up to 7.5 nM concentration.

The results of the PCR arrays exploring changes to cytoskeletal regulators showed that exposure of the HFF-1 cells to NH_2_-QDs caused an increase in CCNA1, CDK5R1, IQGAP2, MYLK2, and WAS genes at all tested concentrations. However, the effects were more significant at the two highest concentrations tested (7.5 and 15 nM) (Fig. 6c).

For COOH-QDs, a set of eleven genes were found to be significantly affected, two of which; MYLK2 and WAS, were also found to be upregulated from exposure to the NH_2_-QDs (Fig. 6d). For the cells exposed to COOH-QDs, these genes were significantly affected along with others, such as ARAP1, CDC42BPA, and CDC42EP2, which were all significantly downregulated at all the tested concentrations.

## Discussion

### Cellular uptake

Though characterisation studies demonstrated a clear difference in fluorescence intensity between the NH_2_- and COOH-QDs, with the latter being much brighter, yet the comparison here is not simply the difference in the uptake level between the two QDs. For the interpretation of the results of confocal microscopy shown in Fig. 1 two different effects need to be discussed. First, a possible difference in cellular internalization of these QDs, as previously demonstrated [20], where negatively charged well dispersed COOH-QDs were shown to be more readily taken up in different cell types compared to the positively charged agglomerated NH_2_-QDs. This however would be in contrast to another study, where positively charged ZnO NPs, prone to agglomeration, were found to be internalized to a higher extent than negatively charged, well dispersed, polymer-coated ZnO NPs [25]. Second, the possibility that the fluorescence properties of the QDs may have changed, leading to changes in intracellular signal not due to changes in QD concentration, but due to fluorescence loss of the QDs upon being localized in acidic endosomes/lysosomes. According to Additional file 1: Figure S11 this effect is stronger for the COOH-QDs than for the NH_2_-QDs, which would explain the loss of intracellular fluorescence over time of cells incubated with COOH-QDs. Fluorescence loss in acidic pH itself may be caused by different mechanisms. Low pH can cause the generation of trap states by partial loss of the ligands shell, which quenches fluorescence. The latter was tested and described in the next paragraph. Third, some of the QDs may have been exocytosed after being endocytosed [26], which also would lead to a decline in the intracellular fluorescence detected over time.

### pH effect on QD stability

Generally, cellular internalization of NPs occurs through endocytic processes [27], during which NP stability may be affected due to changes in the surrounding pH conditions ranging from pH 7.4 representing the extracellular environment, pH 5–6 for late and early endosomes, consecutively to the more acidic pH 4.5 of the lysosomal milieu [28]. It has been shown that various NPs, including QDs, are sensitive to the acidic degrading environment of the lysosomes, resulting in a gradual dissolution of the NPs and release of metal ions, which in this case, would be amongst others, highly toxic Cd^2+^ ions [29]. Therefore, the effect of changing pH levels on NP stability were tested. The results of this test showed no degradation effects for the NH_2_-QDs up to 4 days in all three solutions while the COOH-QDs were found quenched starting from day 2. It is not clear if this observation is due to the high chemical stability of the NH_2_-QDs. One possibility is that these QDs formed large aggregates (as seen in the characterisation results in Additional file 1: Figure S8), which could have sedimented to the bottom of the wells, resulting in an absence of significant signal alteration. Concerning the photophysical properties of the two different types of QDs, within the first day of incubation, there was a significant loss in fluorescence of the (initially bright) COOH-QDs (Additional file 1: Figure S11A) but not for the (already initially weakly fluorescent) NH_2_-QDs (Additional file 1: Figure S11B). The loss in intracellular QD fluorescence from 4 to 24 h after exposure for the COOH-QDs thus could be explained by a possible fluorescence quenching. Partial loss of the ligand shell may have caused the reduction in the fluorescence of the COOH-QDs. However, the NH_2_-QDs were already initially agglomerated, further loss of ligands is thus less likely and thus the initially already weak fluorescence does not decrease further upon incubation.

Alternatively, low pH may lead to corrosion of the QDs, i.e. in their dissolution, leading to the release of free Cd^2+^ ions. In order to investigate the last point, intracellular levels of free Cd^2+^ ions (i.e. Cd^2+^ ions released from internalized QDs) were detected (Additional file 1: Figure S11C), as explained in the next section.

### Changes to QD properties following intracellular uptake

In the intracellular environment the two QDs appear to have undergone some degradation as evidenced with the free Cd^2+^ ions detected. Both QDs were significantly degraded in the cell starting at day 2 for the COOH-QDs and day 3 for the NH_2_-QDs. These data are in line with earlier studies on QDs, where degradation of the QDs typically displays a lag time of one to several days, after which there is a gradual increase in cellular Cd^2+^ levels [21, 30]. Slower release of Cd^2+^ from the NH_2_-QDs may be explained by the fact that they are agglomerated, thus their surface is less accessible. In addition, there is indication that the ZnS shell around the CdSe core is thicker for the NH_2_-QDs than for COOH-QDs (Additional file 1: Table S1), which also may account for the slower release of Cd.

The absolute amount of released Cd^2+^ ions correlates to the number of internalized QDs. However, due to loss in QD fluorescence upon potential partial loss of the ligand shell the data shown in Fig. 1 do not allow us to make a statement about the absolute amount of QDs that has been incorporated by cells. Additional file 1: Figure S11C shows clear release of intracellular Cd^2+^ from internalized QDs. Cadmium is a heavy metal that has been shown to be highly toxic in mammalian cells [31]. Free cadmium ions have also been correlated with toxicity detected in cells exposed to cadmium based QDs [32]. However, Cd^2+^-mediated toxicity would depend on the balance between cell cycle kinetics and degradation kinetics (i.e. the release of Cd^2+^), where toxicity will only occur when the cellular Cd^2+^ concentration exceeds a certain toxic threshold, which may not be the case for highly proliferating cells (i.e. in the limiting case, if cell division were faster than release of Cd^2+^ from internalized QDs) then no Cd^2+^-mediated effects would occur). More subtle, sub-cytotoxic effects will be more easily detected in non-proliferating cells, as they should occur at lower Cd^2+^ levels. This assumption is supported by a previous publication [20], where significant chromosomal damage was detected in HFF-1 cells exposed to 7.5 nM QD concentration of either the NH_2_- or COOH-QDs, conditions under which no acute cytotoxicity was observed.

### Intracellular trafficking of the QDs: confocal microscopy, fSPT, and ICP-MS

In order to gain insight into the kinetics of the uptake of these QDs into HFF-1 cells and to better understand differences in their cellular interaction and consequent effects on cellular homeostasis, we performed confocal microscopy based analysis of cells expressing Lamp1-lysosomal marker and EEA1-early endosomal marker. The positively charged NH_2_-QDs were mainly localized in lysosomal compartments while the COOH-QDs were mostly found to reside in the early endosomes. These results are in good agreement with another study, in which positively charged FeO_x_ NPs with moderate colloidal stability localized only with lysosome, whereas negatively charged FeO_x_ NPs with good colloidal stability first were found in endosomes and the later also in lysosomes [33]. These experiments, however, suffer from the high number of endosomes and lysosomes, which requires high lateral resolution in imaging to delineate all the different cellular organelles. Additionally, all these organelles are dynamic and are in constant movement, where in case of not sufficient lateral resolution some colocalization observed might be accidental due to the close proximity of one passing QD (agglomerate) and cellular organelles. Therefore, in order to overcome these limitations these tests were followed with more precise kinetic studies, which involved live tracking of QDs with a dual colour fluorescence single particle tracking (fSPT) system.

The dynamic, trajectory-based colocalization of the QDs with the stained endosomes or lysosomes was analysed using motion trajectories acquired using the fSPT system via the recorded movies of the identified green and red objects. Algorithms in custom built MatLab software were utilised to perform calculations. The dynamic colocalization coefficient, which detected correlated movement between the two objects, was thus the fraction of trajectories of one fluorescence channel that showed correlated movement with trajectories from the second channel. The fSPT system allows for recording of movies of both, the stained organelles and the QDs under investigation, thereby providing time-dependent, live event information regarding the true colocalization of both components [34]. The results obtained from these experiments were in line with our confocal microscopy results explained above. Results were also in agreement with a study in which (polyethylene-coated) gold (Au) NPs, of high colloidal stability, were passed from small vesicles (<150 nm, such as endosomes) to bigger vesicles (>1000 nm, such as lysosomes), whereas agglomerated Au NPs had their peak inside small vesicles at intermediate incubation times (4 h) [35]. In this cited study both Au NPs were negatively charged.

It has been reported that functionalized NPs are prone to exocytosis [36] which is an important parameter to investigate with NPs that are to be used as imaging contrasts, especially that previous studies on Au NPs have demonstrated differences in intracellular NPs due to exocytosis thus highlighting the importance of duration and concentration of NP exposure for their optimal use for cell labelling [37]. In order to investigate this parameter and to better understand the results of the confocal microscopy analysis (Fig. 1) from this work, where depletion in fluorescence was noted between 4 and 24 h time points following exposure of HFF-1 cells to the COOH-QDs, ICP-MS was conducted. For this purpose, cells were exposed to the QDs for 4 and 24 h, after which the incubation media were removed. Cells were then extensively washed and given fresh QD-free media, after which samples were collected at 0, 30, 60, 120, 240, and 360 min time points to evaluate the presence of free Cd^2+^ ions in the extracellular medium. In the following analysis we are assuming that the detected Cd in the extracellular medium originates from exocytosed QDs (note that ICP-MS measures the elemental amount of Cd, regardless of whether it originates from Cd-based QDs or Cd ions). Also, it is important to note that this assumption excludes QDs sticking to the extracellular membrane, which might not have been removed by the thorough washing steps [38].

The amount of exocytosed QDs should scale with the amount of QDs that have been incorporated by cells before the washing procedure. The higher the exposure concentration of QDs to cells, the higher, thus, the number of exocytosed QDs should be, which was true in these experiments (Fig. 4a). In case one assumes that positively charged agglomerated QDs are internalized to a higher extent than negatively charged well dispersed QDs [25], then the higher number of exocytosed NH_2_-QDs as compared to the COOH-QDs could be understood. It is also worthwhile noting the difference in the size of the COOH- and NH_2_-QDs where the latter is slightly larger where one would assume that some of the additional Cd detected with these NPs is due to the additional Cd atoms present. However, the extent of released Cd ions cannot be justified with only this parameter which makes us assume that there is an effect of NP trafficking also involved in the observed difference. Time dependence of exocytosis of the NH_2_-QDs at low and short exposure condition (2.5 nM, 4 h) follows the trend of average colocalization of these QDs with endosomes (Fig. 4b versus Fig. 3b). As in the colocalization experiments time dependence of exocytosis of NH_2_-QDs does not follow a linear pattern.

### Cytotoxicity studies

The results of the cytotoxicity studies showed a difference in the toxicity profile of the NH_2_-QDs compared to the COOH-QDs where the latter induced more cytotoxicity especially in the form of mitochondrial ROS. Autophagy has been linked to a great variety of NPs [39, 40], and has been associated with different types of QDs in various studies [41, 42], yet no autophagy was found to be induced in these studies. The lack of a clear induction of autophagy is therefore somewhat surprising, but may be due to the nature of the cell type used in the present study. Generally, nanomaterial-induced autophagy is primarily associated with cancer cell types, where in comparative studies, it has been shown that healthy, non-cancerous cell types (such as the ones used in this study) display lower levels of autophagy induction [40, 43]. Similarly, even though ROS has been considered to be a key player in toxicological profile of several types of NPs [44], however, some recent studies have suggested that this view may have been exaggerated, in part due to interactions of the NPs with the most common assays used for ROS detection [45]. In particular for imaging-based experiments, the induction of ROS has not been shown to be clearly predominant with many different types of NPs [46]. Moreover, in some other work we have seen that it is mitochondrial oxidative stress that is associated with the NP induced cellular damage [47].

The lack of any significant effect with the NH_2_-QDs suggests that the mitochondrial ROS induction might be due to the internalization process itself where uptake with these QDs appeared to be less than the COOH-QDs and much more NH_2_-QDs were found to be exocytosed by the cells compared to the COOH-QDs.

### Gene alterations following QD exposure

To support the observations obtained above and to gain more insight into the molecular mechanisms involved in the alterations to the cellular homeostasis and to correlate this to the different trafficking mechanism of the two QDs, the gene expression levels of two key cellular pathways were investigated. The first pathway focuses on genes involved in cellular stress and toxicity, and can be seen as an overview of cellular homeostasis. Different sets of genes were up- or down-regulated following 24 h exposure to NH_2_- or COOH-QDs. Cell exposed to NH_2_-QD resulted the upregulation of CCL2, IL1A, IL1B, IL6, IL8, and TNFα genes, all of which are involved in the induction of inflammatory responses [48]. Similar effects have been reported following exposure to various NPs. For example, exposure of leukocytes, monocytes, and macrophages isolated from human blood, to polystyrene NPs, resulted in an increase in phagocytosis due to the presence of the NPs [49]. In contrast, exposure to COOH-QDs resulted in a decrease in an array of genes mainly involved in cellular hypoxia. This finding is in line with earlier findings, where the involvement of genes linked to hypoxia have been associated with cellular NP toxicity [50]. The induction of high levels of mitochondrial metabolism, as indicated by the induction of mitochondrial ROS, may result in an artificial hypoxia-like scenario. Although the level of available oxygen is sufficient for basal cellular metabolism, the persistent higher metabolism results in higher energy demands, which may not always be met by the overproducing mitochondria. This “*lack of energy*” therefore will be highly similar to the typical scenario of low oxygen consumption, resulting in alterations to the expression levels of genes typically associated with hypoxia. The occurrence of hypoxia-like processes is interesting, because hypoxia is a main feature of tumor cells resulting in resistance to cancer therapeutic agents [51]. It has been reported that some of the primary adaptive responses to hypoxia include the expression of genes involved in angiogenesis, such as the vascular endothelial growth factor A (VEGFA) gene and the SLC2A1 gene responsible for the metabolic adaption of cells [52]. Previous reports have suggested that the inhibition of these genes could lead to killing of tumor cells or the suppression of resistance to cancer therapeutic agents [51]. This raises the question of whether such NPs could be used for therapeutic applications. Interestingly, no changes were noted in this array from both QDs for the genes involved in oxidative stress which is consistent with our ROS results presented above.

Next to cellular stress and toxicity responses, we also looked into analysis of the cytoskeletal regulator pathway genes. Array results showed that exposure of cells to NH_2_-QDs induced an increase in the levels of a few genes that are involved in cell mobility and migration. Some of these genes, such as IQGAP2, which effects cellular morphology by regulating the actin cytoskeleton by interacting with cytoskeletal components, cell adhesion, and cell signaling molecules [53–55]. This gene has been implicated in invasion and metastasis of cancer cells [55]. MYLK2, and WAS genes, which were also significantly upregulated in NH_2_-QD treatments, are involved in the trafficking of molecules into the cell [56, 57]. Exposure to the COOH-QDs resulted in an array of upregulated and downregulated genes. The most notable ones were ARAP1, CDC42BPA, and CDC42EP2 which are genes involved in forming cell projections and their downregulation is in line with the high-content imaging studies, where at higher COOH-QD concentrations cells were less spread resulting in a lower cell surface area. The deformation of cellular cytoskeleton networks by various NPs is also in line with various other reports, where, in particular at higher NP concentrations, clear deformations of actin and tubulin cytoskeleton have been observed, which could result in secondary effects like altered cellular mobility and migration capacities [58].

## Conclusions

**Fig. 7:**
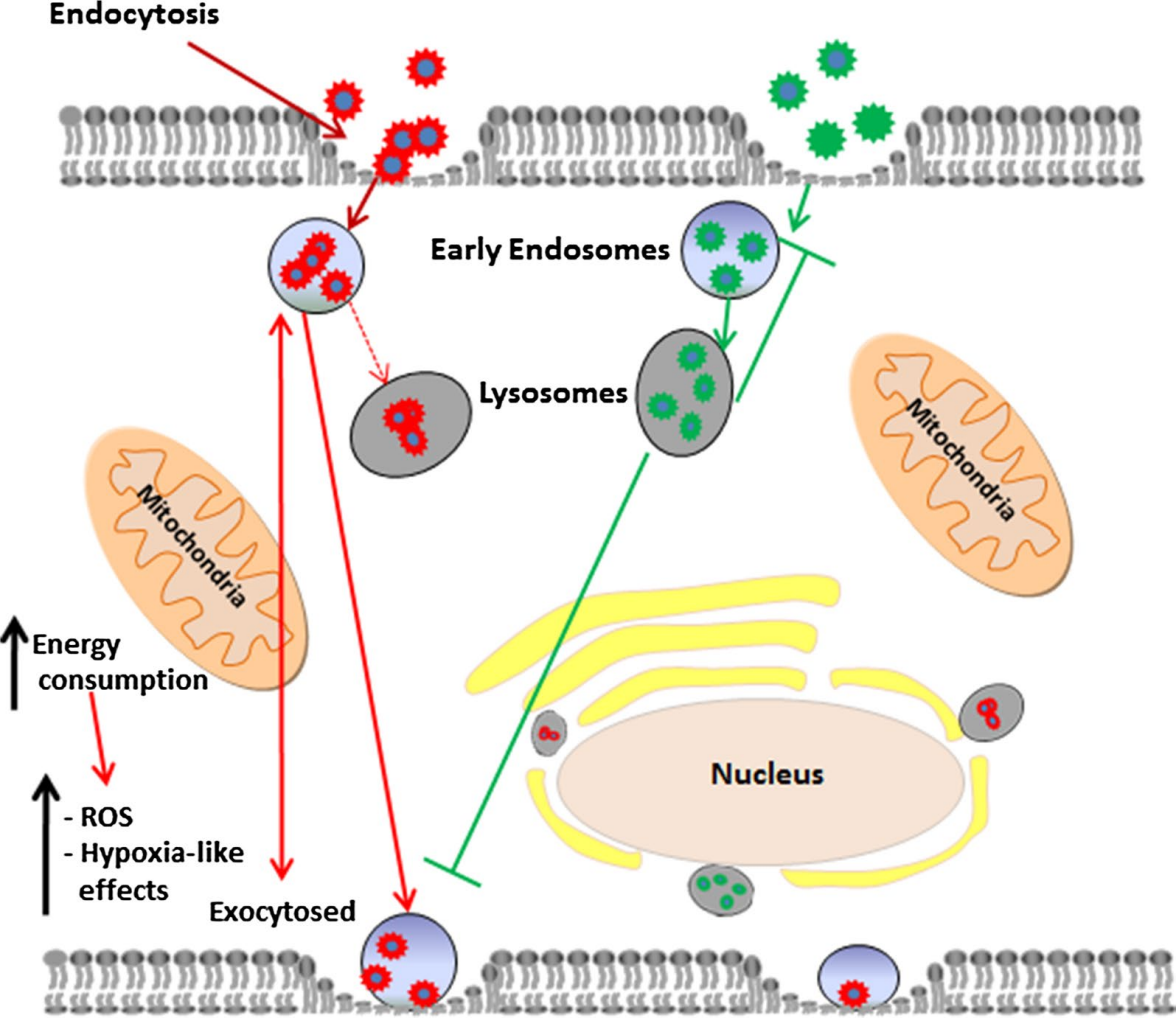
A figure illustrating the hypothesis that the kinetics of nanoparticle uptake and intracellular processing can vary due to their physico-chemical properties resulting in differences in their toxicity profiles

Most NP studies consider physico-chemical properties and their correlation to either kinetics, or toxicity. The present work reveals the importance of understanding how the cell interacts with NPs from a kinetic and mechanistic point of view and then how to interpret these observations to NP properties in an effort to elucidate the differences observed in toxicity and gene alteration results between different NPs. Upon exposing human fibroblasts to two types of QDs, one with COOH moieties, which was well dispersed, and the other with NH_2_ moieties, which was agglomerated, the toxicological profile for these QDs was different. The state of agglomeration turned out to be a very relevant physicochemical parameter describing the difference between both types of QDs. The latter clearly had an effect on the process by which the cells trafficked these NPs thus resulting in different effects on cellular homeostasis. The cellular uptake was studied at different time points, where clear differences were observed. NH_2_-QDs were taken up by the cells rather quickly, but soon resulted in a steady-state level, after which no additional uptake was observed, and were eventually transferred to the lysosomal compartment (Fig. 3b). COOH-QDs followed a different pathway, where they were internalized at a high rate which persisted over at least 6 h (Fig. 3c). There was only a minimal transfer of COOH-QDs to the lysosomal compartment. Generally, both QDs but more so with the NH_2_-QDs perinuclear localization of the NPs was noted which could be due to the residence of more acidic lysosomes that are performing degradation process in that region [59]. Although no acute cytotoxicity was observed for either of the two QD types under the conditions used, the differences in cellular internalization however resulted in variations in their stress response profile, where high-content imaging and gene expression studies revealed the induction of mitochondrial ROS, cytoskeletal remodelling, and hypoxia-like cellular responses from exposure to the COOH-QDs, which could all be linked to higher energy demands. A hypothetical sketch is shown in Fig. 7.

Together, these data reveal that though the two QDs differed in physico-chemical properties they were internalised by the cells to a similar extent. Differences in their uptake kinetics, however, appear to be accountable for the significant changes discovered in their toxicity and gene expression profiles.

## Methods

### Cell culture

Human foreskin fibroblasts HFF-1 (ATCC Manassas, VA) cells were cultured in Dulbecco’s Modified Eagle’s Medium (DMEM) in the presence of 15% foetal bovine serum (FBS, Gibco, Life Technologies, Belgium). Cells were incubated at 37 °C and 5% CO_2_ and sub-cultured every third day. All cellular treatments were at 0, 2.5, 5, 7.5, 10, and 15 nM concentrations. All experiments were performed in triplicates.

### Quantum dot nanoparticles

Both QDs used in this work were commercial products. CdSe/ZnS core/shell fluorescent NPs with NH_2_ (Cytodiagnostics, Canada) and COOH (Invitrogen, UK) functional ligands were used. Details about the structure of the semiconductor part as well as the surface chemistry are not disclosed by the providers. These QDs had emission maxima of 664 nm (nominally 665 nm) and 585 nm (nominally 590 nm). These QDs have been previously thoroughly characterised (please see Additional file 1 for details) [18–20]. QD concentrations for exposure experiments were based on the concentrations of the QDs stocks as given by the suppliers. Cellular exposure stocks were prepared by diluting the QDs in sterile phosphate buffered saline (PBS). All concentration suspensions were vortexed for 30 s prior to addition to the cell culture. Exposure to QDs were for 4 or 24 h.

### QD uptake studies

Confocal microscopy and ICP-MS analyses were conducted to examine QD uptake into HFF-1 cells following 4 and 24 h exposure. Details can be found in Additional file 1.

### Analysis of photo-stability of the QDs

The effect of the lowered pH levels in the intracellular environment on the photo-stability of the QDs was determined by examining the possible effects of altered pH levels. Experiments were performed as previously described [21]. More details on the methods used can be found in Additional file 1.

### Cellular interaction with QDs

The consequence of cell QD interaction in terms of the generation of cytoplasmic and mitochondrial reactive oxygen species (ROS), the level of the lipidated LC3 protein (marker for autophagy), and cytoskeletal changes were investigated using high-content image analysis as detailed previously [14]. A detailed experimental section of these studies can be found in Additional file 1.

### QD tracking studies

Single particle tracking (SPT) and confocal microscopy based analyses were conducted to track NH_2_- or COOH-QDs in the intracellular environment, and to determine their colocalization with endosomes or lysosomes. Full details of the methodology can be found in Additional file 1.

### Inductively coupled plasma mass spectrometry (ICP-MS)

Inductively coupled plasma mass spectrometry (ICP-MS) was conducted in order to determine the number of QDs excreted by the cells. For this end, cells were labelled with QDs at 2.5, 7.5, and 15 nM concentrations for 4 and 24 h. Cells were then washed three times with sterile PBS and supplemented with fresh culture media. Samples were collected from the culture supernatant at 0, 30, 120, 240, and 360 min. The amount of elemental cadmium and selenium in the samples was determined using ICP-MS (see Additional file 1: Section 4 for more details).

### Gene expression studies

Two important human gene expression pathways, the human cytoskeletal regulatory and the cellular stress and toxicity pathways, were investigated using real time polymerase chain reaction (RT-PCR) arrays as described previously [14]. Briefly, 1.5 × 10^5^ cells/mL were allowed to settle overnight, followed with incubation with 0, 2.5, 7.5, and 15 nM NH_2_- or COOH-QDs for 24 h (see Additional file 1: Section 9 for more details).

### Statistical analysis

All data are expressed as the mean ± standard deviation (SD), unless indicated otherwise. All experiments, except the PCR arrays, were analysed using the One Way Anova statistical method. Significance in the PCR arrays was determined based on twofold change from the control ΔΔCt value.

## Additional file

**Additional file 1.** Nanoparticles characterisation, additional materials and methods and data.
