# Supplementary material for: The role of intracellular trafficking of CdSe/ZnS QDs on their consequent toxicity profile
Source: J Nanobiotechnology. 2017 Jun 15;15:45. doi: 10.1186/s12951-017-0279-0 (PMC5472855; doi:10.1186/s12951-017-0279-0)
Supplement: Supplementary file 1 — Additional file 1. Nanoparticles characterisation, additional materials and methods and data. [file 12951_2017_279_MOESM1_ESM.docx]

**The role of intracellular trafficking of CdSe/ZnS QDs on their consequent toxicity profile**

Bella B. Manshian^1,2,*^, Thomas F. Martens^3,4^, Karsten Kantner^5^, Kevin Braeckmans^3,4^, Stefaan C. De Smedt^3^, Jo Demeester^3^, Gareth J.S. Jenkins^2^, Wolfgang J. Parak^5,6^, Beatriz Pelaz^5^, Shareen H. Doak^2^, Uwe Himmelreich^1^, Stefaan J. Soenen^1^.

^1^ Biomedical NMR Unit/MoSAIC, KULeuven Campus Gasthuisberg, Radiology Department, Herestraat 49, Leuven B3000, Belgium.

^2^ Institute of Life Science, College of Medicine, Swansea University, Singleton Park, Swansea SA2 8PP, UK.

^3^ Faculty of Pharmaceutical Sciences, Ghent University, Harelbekestraat 72, B9000 Ghent, Belgium.

^4^ Center of Nano- and Biophotonics, Ghent University, Harelbekestraat 72, B9000 Ghent, Belgium.

^5^ Philipps University of Marburg, Renthof 7, D35032 Marburg, Germany

^6^ CICBiomagune, San Sebastian, Spain

*Corresponding author: Dr. Bella Manshian

Email: [bella.manshian@kuleuven.be](mailto:bella.manshian@kuleuven.be)

Tel: 0032 16 23 30 58

Fax: 0032 16 34 59 91

**Keywords**: Quantum dot NPs, intracellular localization, endosomal uptake, gene alterations, nanotoxicity

**Additional Materials and Methods**

**1. QD characterization studies**

**1.1 Transmission Electron Microscopy**

**1.2 Concentration determination of the samples**

**1.3 Comparison of emission properties of QDs**

**1.4 Colloidal stability of the QDs in water and in complete cell culture media**

**1.5 Colloidal stability of the QDs *versus* NaCl**

**1.6 Summary**

**2. Effect of pH on QD fluorescence stability**

**3. Determination of free cellular cadmium ions**

**4. Evaluation of cellular excretion of QDs**

**5. High content cellular image analysis**

**5.1 Cytoskeletal changes**

**5.2 InCell ROS analysis**

**5.3 InCell LC3 analysis**

**6. Confocal microscopy of QD uptake and changes in tubulin fibers**

**7. Endosomal / lysosomal and single particle tracking**

**8. Colocalization of QDs with lysosomes or endosomes *via* confocal microscopy**

**9. Gene expression studies**

**10. Supporting references**

**1. QD characterization studies**

**1.1 Transmission Electron Microscopy**

To determine the core size of the quantum dots (QDs), *i.e.* the inorganic CdSe/ZnS part excluding the organic surface capping, transmission electron microscopy (TEM) images were collected. The purchased QDs were diluted in water and one drop was deposited onto a copper grid. The size of the QD cores was determined using the free software Image J. TEM images and histograms are shown in **Figure S1**. The diameter d_c_ of the cores was determined as 4.6 ± 0.5 nm for the carboxyl-QDs (QD-) and 6.9 ± 0.9 nm the amine-QDs (QD+). It is worth to mention that the monodispersity of the QDs+ was not very good, typically the presence of big nanoparticles aggregates was observed (Figure S1, panel d).

**Figure S1**. TEM images and histograms of a), c) and e) QD-; b), d) and f) for QD+.

**1.2 Concentration determination of the samples**

Inductively coupled plasma mass spectrometry (ICP-MS) was used to determine the concentration of the QDs, and to correlate it with both, absorbance and emission spectra. To do so, the as received samples of QDs+ and QDs- were digested for at least 4 h with freshly prepared aqua regia at room temperature and diluted in 2% HCl prior to measuring them *via* ICP-MS. The content of cadmium (C_Cd_), zinc (C_Zn_), and selenium (C_Se_) was determined in terms of mass concentrations, *i.e.* mass of element per volume of solution. Then, the total mass concentration of QDs (C_QD_) was determined as C_QD_ = C_Cd_ + C_Zn_ + C_Se_ + C_S_. The sulphur content, C_S_ was determined by considering a molar ratio 1:1 to the Zn content (i.e. in the sample the same number of S atoms as Zn atoms is assumed; for the mass concentrations the atomic masses of S and Zn are used as scaling factors). In this way the mass fractions of the CdSe core and the ZnS shell of the inorganic parts of the QDs were determined as f_CdSe_ = (C_Cd_ + C_Se_)/C_QD_) and f_ZnS_ = ((C_Zn_+C_S_)/C_QD_), (**Table S1**).

| ***Sample*** | ***C_Zn_ [ppb]*** | ***C_Se_ [ppb]*** | ***C_Cd_[ppb]*** | ***C_S_ [ppb]*** | ***C_QD_ [ppb]*** | ***f_CdSe_*** | ***f_ZnS_*** |
| --- | --- | --- | --- | --- | --- | --- | --- |
| QDs+ | 1338608 | 313464.7 | 807472.9 | 649022 | 3108567.5 | 0.36 | 0.64 |
| QDs- | 714973 | 233605.9 | 1717182.8 | 346653.6 | 3012415.3 | 0.65 | 0.35 |

**Table S1**: Summary of the inorganic content in QDs+ and QDs- as determined by ICP-MS. C_X_ = 1 ppb (part per billion) refers to 10^-9^ g of element X in 1 g of water, *i.e.* in 1 mL of water. C_QD_ [g/mL] = 10^-9^⋅C_QD_ [ppb].

To convert element (Cd, Se, Zn, S) concentrations to QD concentrations the QDs were considered as spherical particles. Then, the mass of an individual QD, m_QD_, (note that the organic surface capping is excluded), was calculated after determining the volume, V_QD_, and the density of a single QD, ρ_QD_. To determine the density, we made an approximation by taking the densities of the CdSe core and the ZnS shell, weighted by their mass fractions (**Equation S1**).

ρ_QD_ = ρ_CdSe_·f_CdSe_ + ρ_ZnS_·f_ZnS_

Using ρ_CdSe_ = 5.82 g/cm^-3^, ρ_ZnS_ = 4.09 g/cm^-3^.

The volume of an individual spherical QD (V_QD_ = (4/3)·π·(d_c_/2)^3^), was calculated using the core diameter as determined by TEM (d_c_, **Figure S1**). Then, the mass m_QD_ of one QD (m_QD_ = V_QD_·ρ_QD)_ multiplied by Avogadro´s number, N_A_, yields the molar mass, M_QD_, of the QD (**Equation S2**).

M_QD_ = V_QD_·ρ_QD_·N_A_

The molar concentration c_QD_ [mol/L] can then be calculated from the mass concentration C_QD_ [g/L] using the molar mass M_QD_ [g/mol] as scaling factor.

c_QD_ = C_QD_/M_QD_

A summary of the calculated values for both samples is presented in Table S2. Note that for the studies investigating the colloidal stability of the QDs the concentrations c_QD_ as determined by ICP-MS were used. For the rest of the study the concentrations c_QD_ as provided by the suppliers were used.

| ***Sample*** | ***ρ_QD_ [g/cm^-3^]*** | ***V_QD_ [nm^-3^]*** | ***m_QD_ [g]*** | ***M_QD_ [g/mol]*** | ***C_QD_ [mg/mL]*** | ***c_QD_ [M]*** |
| --- | --- | --- | --- | --- | --- | --- |
| QDs+ | 4.71 | 172 | 8.10 · 10^-19^ | 4.88·10^5^ | 3.11 | 6.37·10^-6^ |
| QDs- | 5.21 | 49.9 | 2.60 · 10^-19^ | 1.57.·10^5^ | 3.01 | 1.92·10^-5^ |

**Table S2**: Summary of the calculated values for QDs- and QDs+, the measured QD mass concentration of C_QD_ in mg/mL, and the QD concentration c_QD_ [M].

**1.3 Comparison of emission properties of QDs**

The emission efficiency of both QDs was compared using two solutions with approximately the same known concentration, and using the same excitation wavelength (ε_ex_ =450 nm). As emission intensity, I, at the maximum for each sample was selected, λ_em, QDs+_ = 664 nm and ε_em, QDs-_ =585 nm for the QDs+ and QDs- respectively. The emission intensities were directly related, resulting in that the QDs- are *ca.* 1400 times more bright than the QDs+ (**Table S3** and **Figure S2**).

| **Sample** | ***c_QD_* [M]** | ***I* [a.u.]** | ***R_IQDs-/IQDs+_*** |
| --- | --- | --- | --- |
| QDs- | 1.5·10^-8^ | 5.53·10^8^ | 1441 |
| QDs + | 1.5·10^-8^ | 3.84·10^5^ |  |

**Table S3**: Summary of the measured intensity emissions for two samples of QDs with equal concentration, and the ratio of the intensity of emission among them, R_IQDs-/IQDs+_.

**Figure S2**. Emission spectra of the QDs, as measured in water, using as excitation wavelength 450 nm.

**1.4 Colloidal stability of the QDs in water and in complete cell culture media**

The stability of the QDs was studied by dynamic light scattering (DLS) after diluting the QDs in complete cell culture media with different percentages of fetal bovine serum (FBS). Concentrations of QDs were c_QD_ = 237 and 155 nM for QDs- and QDs+, respectively. In the case of the negatively charged QDs, the QDs exhibited a good colloidal stability and values of hydrodynamic diameter as recorded in medium and in water were in agreement. The positively charged QDs in contrast had much bigger hydrodynamic diameter in cell medium than in water, and thus are agglomerated. Results are summarized in **Table S4** and in **Figures S3 and S4.** Zeta potential (ζ) measurements confirmed the sign of charge of the QDs.

| **Sample** | ***d_h(I)_* [nm]** | ***d_h(V)_* [nm]** | ***d_h(N)_* [nm]** | ***PdI*** | ***ζ [mV]*** |
| --- | --- | --- | --- | --- | --- |
| QDs- water | 42.44 ± 21.70 | 23.75 ± 10.66 | 17.48 ± 5.33 | 0.230 | -46.9± 10.7 |
| QDs - media | 45.02 ± 22.32 | 26.08 ± 11.43 | 19.30± 5.80 | 0.303 | - |
| QDs+ water | 15.94 ± 6.44 | 10.72 ± 3.99 | 8.43± 2.36 | 0.445 | 44.0 ± 8.8 |
| QDs+ media | 410.8 ± 94.72 | 437.9 ± 1.58 | 375.4 ±94.89 | 0.405 | - |

**Table S4**. Mean hydrodynamic diameter as derived from number (*d_h(N)_*), volume (*d_h(V)_*), and intensity (*d_h(I)_*) distributions *N(d_h_)*, and polydispersity index (*PdI*) for the QDs+ and QD- in cell culture media with 10 % of FBS.


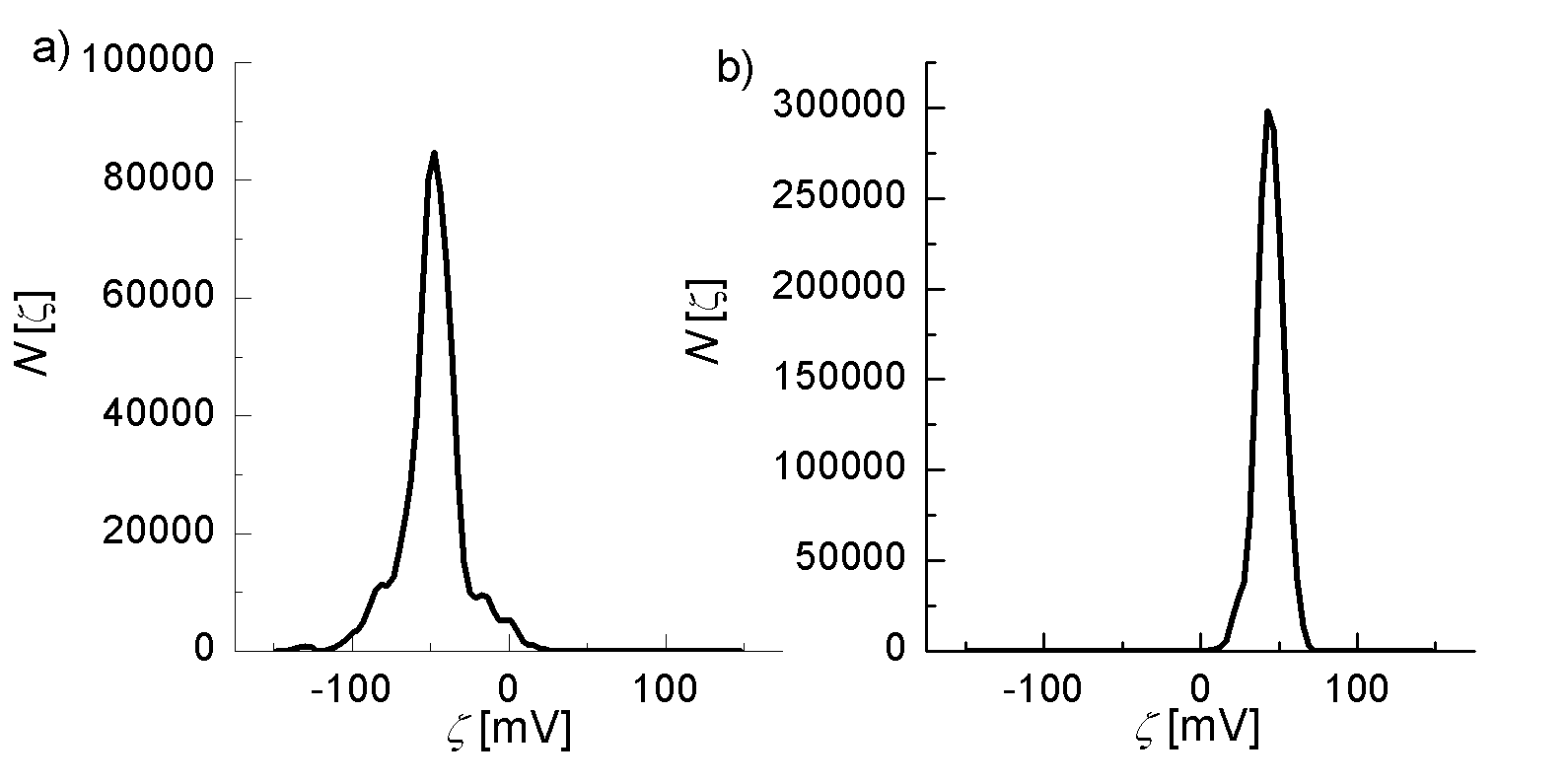


**Figure S3**. Zeta-potential distributions for a) QDs- and b) QDs+ in water.


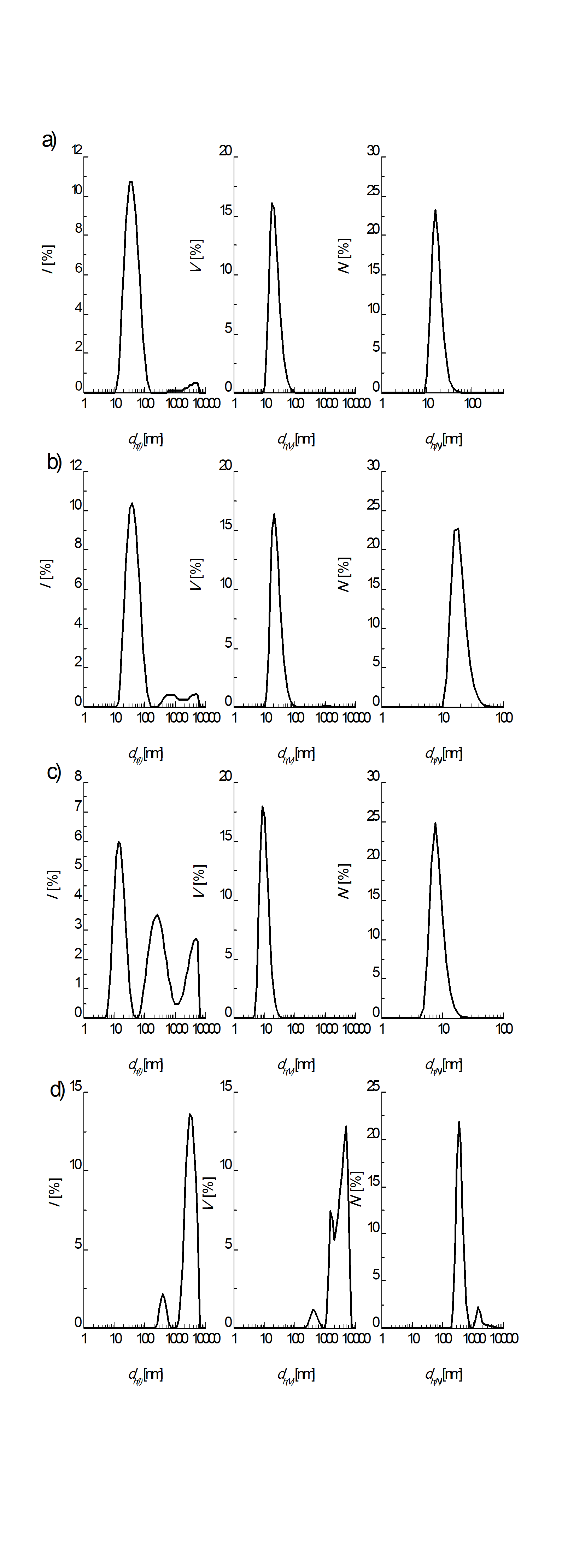


**Figure S4**. Intensity, volume and number distributions of hydrodynamic diameters of a) QDs- in water, b) QDs- in complete cell culture media, c) QDs+ in water and d) QDs+ in complete cell culture media.

**1.5 Colloidal stability of the QDs *versus* NaCl**

The colloidal stability of the QDs against NaCl was tested using both, DLS and UV/Vis absorption spectroscopy. Different concentrations of NaCl (c_NaCl_), were tested at time 0 (upon the direct addition of NaCl) and after 24 h of incubation. The concentrations used were c_QD_ = 0.693 µM and 1.21 µM for QDs- and QDs+, respectively. As it can be seen in **Figure S5**, the aggregation of the QDs+ can be detected just by naked eye, for concentrations equal or higher than 300 mM of NaCl. To collect the DLS and UV/Vis absorption data, the QD solutions were shaken to redisperse the sedimented / aggregated QDs.


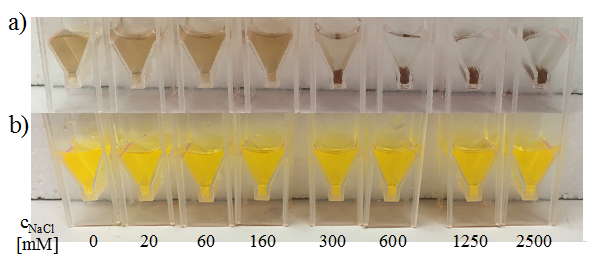


**Figure S5**. Stability of QDs against NaCl after 24 h of a) QDs+ and b) QDs-.

UV/Vis absorption spectra confirmed the above observation that QDs+ aggregate with salt concentrations from 300 mM. For QDs-, no evidence of aggregation was observed using this technique (**Figure S6 and S7**).

**Figure S6**. Absorption spectra A(λ) of QD+ against different c_NaCl_ at a) t = 0 and b) t = 24 h.


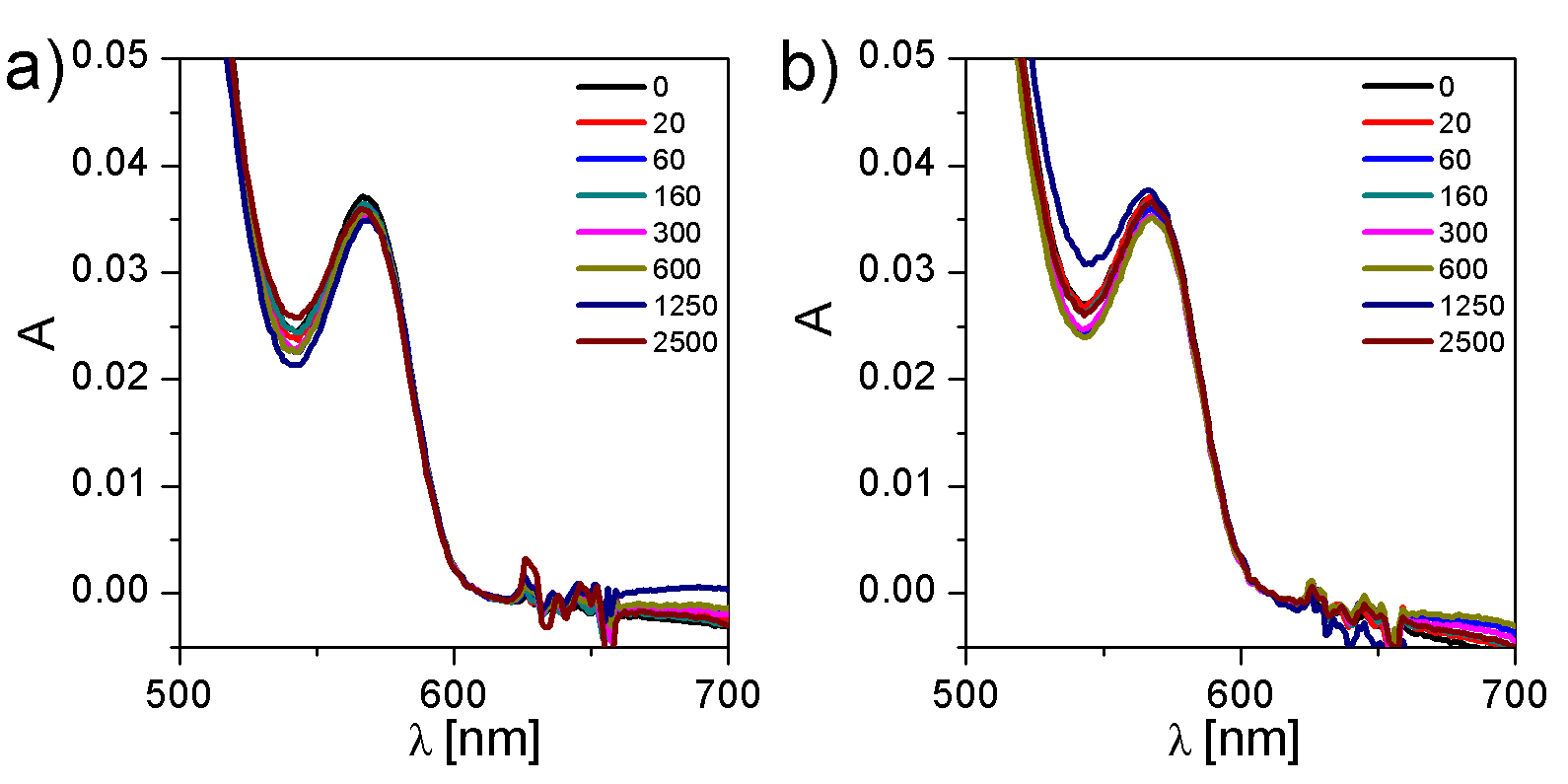


**Figure S7**. Absorption spectra of QD- against different c_NaCl_ at a) t = 0 and b) t = 24 h.

DLS data confirmed the results obtained by UV/Vis absorption spectra and proved that QDs+ after 24 h in a solution of 160 mM NaCl (a value close to that used in cell culture) are aggregated. On the other hand, QDs- remain stable up to NaCl concentrations of 600 mM. Partial aggregation occurs at 1250 and 2500 mM (**Figures S8, S9, S10 and Tables S5, S6**).

**Figure S8**. Hydrodynamic diameter of QDs- and QDs+ *versus* NaCl of different concentrations after 0 and 24 h, as obtained from number distributions recorded by DLS.

| **t [h]** | ***c_NaCl_* [mM]** | ***d_h(N)_* [nm]** | ***PdI*** | **t [h]** | ***c_NaCl_* [mM]** | ***d_h(N)_* [nm]** | ***PdI*** |
| --- | --- | --- | --- | --- | --- | --- | --- |
| 0 | 0 | 7.76 ± 2.35 | 0.492 | 24 | 0 | 7.02 ± 2.24 | 0.676 |
| 0 | 20 | 6.10 ± 2.00 | 0.761 | 24 | 20 | 8.57 ± 2.72 | 0.748 |
| 0 | 60 | 11.31 ± 3.40 | 0.504 | 24 | 60 | 15.45 ± 4.74 | 0.675 |
| 0 | 160 | 82.94 ± 33.73 | 0.563 | 24 | 160 | 59.08 ± 7.71 | 0.626 |
| 0 | 300 | 77.54 ± 58.86 | 0.407 | 24 | 300 | 88.82 ± 20.62 | 0.486 |
| 0 | 600 | 858.6 ± 386.9 | 0.511 | 24 | 600 | 1307 ± 263.3 | 0.487 |
| 0 | 1250 | 177.5 ± 62.38 | 0.497 | 24 | 1250 | 1770 ± 392.6 | 0.330 |
| 0 | 2500 | 170.7 ± 42.73 | 0.515 | 24 | 2500 | 2189 ± 533.7 | 0.151 |

**Table S5**. Mean hydrodynamic diameter in number (*d_h(N)_*), and polydispersity index (*PdI*) for hydrodynamic diameter of QDs+ in NaCl of different concentrations at time 0 and 24 h.

| **t [h]** | ***c_NaCl_* [mM]** | ***d_h(N)_* [nm]** | ***PdI*** | **t [h]** | ***c_NaCl_* [mM]** | ***d_h(N)_* [nm]** | ***PdI*** |
| --- | --- | --- | --- | --- | --- | --- | --- |
| 0 | 0 | 18.51 ± 5.41 | 0.188 | 24 | 0 | 17.74 ± 5.30 | 0.183 |
| 0 | 20 | 17.43± 5.09 | 0.245 | 24 | 20 | 16.55 ± 5.01 | 0.239 |
| 0 | 60 | 17.58 ± 5.02 | 0.206 | 24 | 60 | 17.21 ± 4.95 | 0.233 |
| 0 | 160 | 16.79 ± 5.02 | 0.246 | 24 | 160 | 18.16 ± 5.03 | 0.197 |
| 0 | 300 | 18.03 ± 5.13 | 0.163 | 24 | 300 | 18.85 ± 5.41 | 0.171 |
| 0 | 600 | 19.46± 6.88 | 0.194 | 24 | 600 | 25.60 ± 7.75 | 0.181 |
| 0 | 1250 | 48.62 ± 16.34 | 0.174 | 24 | 1250 | 62.17 ± 22.26 | 0.172 |
| 0 | 2500 | 95.18 ±76.50 | 0.287 | 24 | 2500 | 1426 ± 245.7 | 0.227 |

**Table S6**. Mean hydrodynamic diameter in number (*d_h(N)_*), and polydispersity index (*PdI*) for hydrodynamic diameter of QDs- in NaCl of different concentrations at time 0 and 24 h.


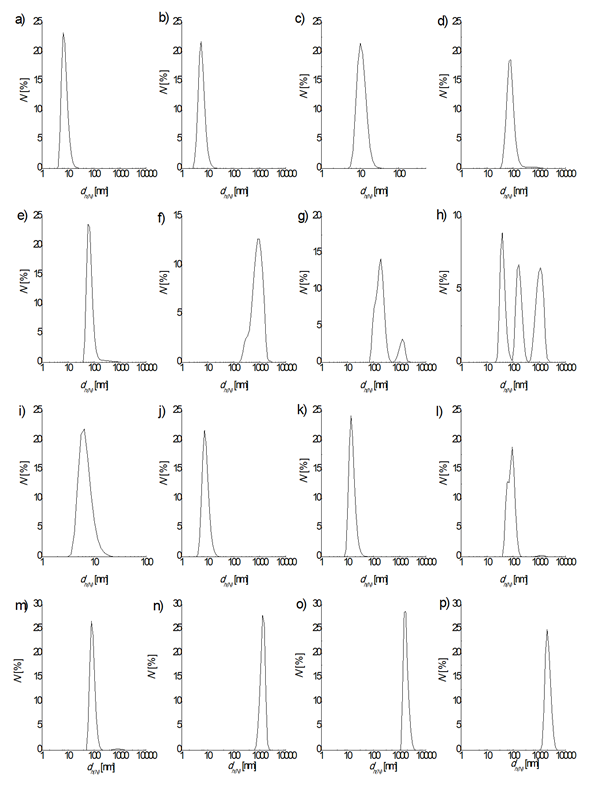


**Figure S9**. Number distribution of the hydrodynamic diameters of QD+ against 8 different concentrations of NaCl (c_NaCl_): a) 0 mM, b) 20 mM, c) 60 mM, d) 160 mM, e) 300 mM, f) 600 mM, g) 1250 mM and h) 2500 mM at t=0; and i) 0 mM, j) 20 mM, k) 60 mM, l) 160 mM, m) 300 mM, n) 600 mM, o) 1250 mM and p) 2500 mM at t=24 h.


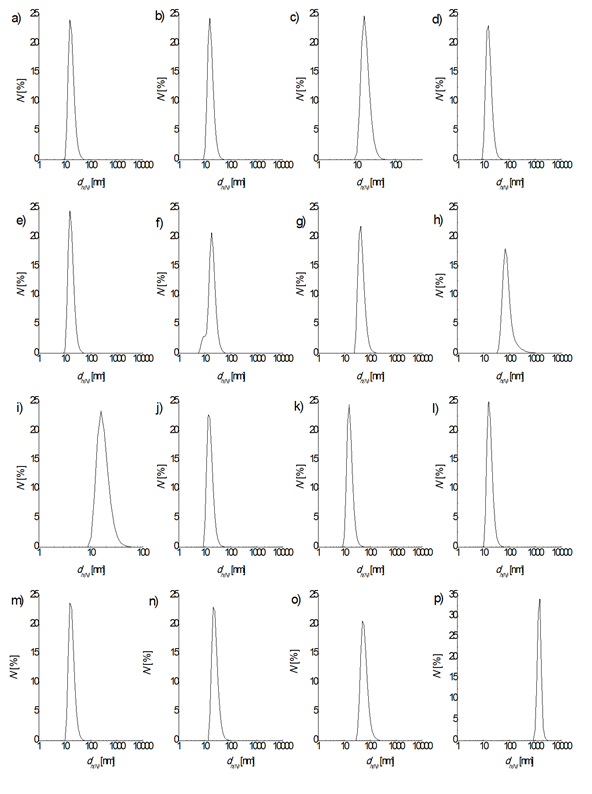


**Figure S10**. Number distributions of the hydrodynamic diameters of QD- against 8 different concentrations c_NaCl_ a) 0 mM, b) 20 mM, c) 60 mM, d) 160 mM, e) 300 mM, f) 600 mM, g) 1250 mM and h) 2500 mM at t=0; and i) 0 mM, j) 20 mM, k) 60 mM, l) 160 mM, m) 300 mM, n) 600 mM, o) 1250 mM and p) 2500 mM at t=24 h.

**1.6 Summary**

| ***Sample*** | ***d_c_ [nm]*** | ***f_CdSe_*** | ***f_ZnS_*** | ***λ_em_* [nm]** | **I(λ_em_) [a.u.]** | **ζ^H2O^ [mV]** | **d_h_^H2O^ [nm]** | **d_h_^med^ [nm]** |
| --- | --- | --- | --- | --- | --- | --- | --- | --- |
| QDs+ | 6.9 ± 0.9 | 0.36 | 0.64 | 664 | 1 | +44.0 ± 8.8 | 8.43± 2.36 | 375.4 ±94.89 |
| QDs- | 4.5 ± 0.5 | 0.65 | 0.35 | 585 | 1441 | -46.9± 10.7 | 17.48 ± 5.33 | 19.30± 5.80 |

As the used QDs are commercial products, their precise composition is not known, as it is not disclosed by the vendors. In case of the QDs+ (Cytodiagnostics, Canada) the material data safety sheet indicated a CdS_x_Se_1-x_/ZnS structure. On top of the ZnS shell there is a layer only referred to as "Advanced Isolation Mechanism (AIM) Technology". In the case of the QDs- (Invitrogen, UK) according to the manufacturer they "are made from nanometer scale crystals of a semiconductor material (CdSe), which are shelled with an additional semiconductor layer (ZnS).

**Table S7**. Summary of characterization data of QDs- (Invitrogen, UK) and QDs+ (Cytodiagnostics, Canada). *d_c_* is the diameter of the inorganic core (CdSe/ZnS), as measured with TEM. *f_CdSe_* and *f_ZnS_* describe the atomic fraction of the inorganic CdSe core and the ZnS shell, respectively. λ_em_ is the emission wavelength of the QDs. I(λ_em_) is the emission intensity at the wavelength of emission. ζ^H2O^ is the zeta potential as determined in MilliQ water. d_h_^H2O^ and d_h_^med^ are the mean values of the hydrodynamic diameters as determined from the number distribution for QDs dispersed in water and cell medium, respectively.

The emission data λ_em_ can be related to the diameter d_c_ of the inorganic CdSe/ZnS core. Yu *et al* relate the diameter of a CdSe QD d_CdSe_^λ^ to its emission wavelength λ_em_ [[1](#_ENREF_1)]. For QDs+ an assumed CdSe diameter of 6.9 nm would correspond to an emission wavelength of 640 nm. Growth of a ZnS shell shifts the emission wavelength to higher values, typically in the range of 10 nm- 30 nm. Thus, the optical data are in agreement with the TEM data. The core of the CdSe core will be < 6.9 nm, the ZnS shell add up to a final diameter of 6.9 nm, resulting in an emission at 664 nm. In case of the QD- data the optical data do not match the TEM data. The TEM data of the CdSe/ZnS QDs provide much higher diameters than the size of the CdSe part should be according to the optical measurements. This might be explained by the asymmetric shape of these QDs.

| QDs+ | ***λ_em_* [nm]** | ***d_CdSe_^λ^* [nm]** | QDs- | ***λ_em_* [nm]** | ***d_CdSe_^λ^* [nm]** |
| --- | --- | --- | --- | --- | --- |
|  | 664 | 9.1 |  | 585 | 4.0 |
|  | 640 | 6.9 |  | 671 | 9.8 |

**Table S8**. Calculation of the diameter of CdSe d_CdSe_^λ^ in dependence of the emission wavelength of CdSe λ_em_, as calculated by the formula from Yu *et al* [[1](#_ENREF_1)]. A ZnS shell will increase the diameter and also red-shift the wavelength of the emission peak.

**2. Effect of pH on QD fluorescence stability**

The effect of different pH levels on the fluorescence of these QDs was investigated as described previously [[2](#_ENREF_2), [3](#_ENREF_3)]. In short, the QDs were incubated in 15% FBS mixed with 10 µM citrate-containing phosphate-buffered saline, whereby the pH levels were adjusted to 7.4, 5.5, and 4.5. QD suspensions were prepared at cQD = 2.5, 5, 5.5, 10, and 15 nM concentrations in 100 μL total volume. The QD concentrations were determined from the concentrations of the QD stock solutions as provided by the suppliers. QDs were incubated with the different media in black 96 well plates (Greiner Bio One BVBA, Belgium). All experiments were conducted in triplicate and were accompanied by a negative control. Fluorescence measurements were taken with an Omega multiwell plate reader (BMG Labtech, Belgium) on days 1, 2, 3, 4, and 5 post preparation of the suspensions. Even though these experiments were conducted at all the tested concentrations, for conciseness, the data shown here present only data for one representative concentration (c_QD_ = 15 nM) (**Figure 11A, B**).


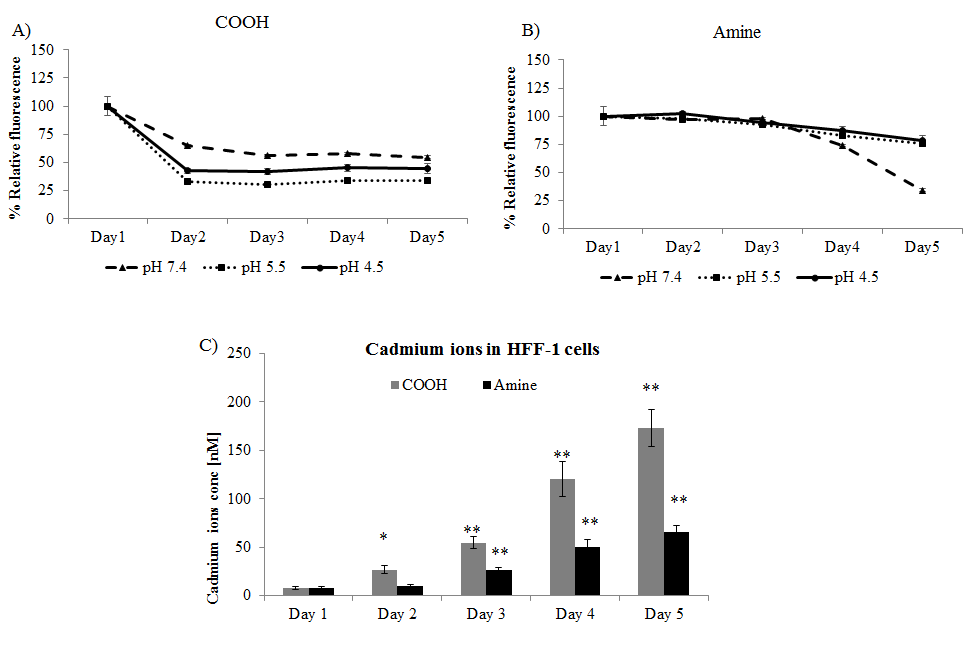


**Figure S11**. A, B) The fluorescence of the QDs is reduced over time. C) Following a prolonged incubation of 2 or more days, clear degradation of the carboxylated QDs (QDs-) can be seen in the form of free cadmium ions detected in solution. The concentration reported here is the concentration of free Cd. The determination of free Cd is described in the next section (though in this paragraph it was carried out without cells). Data are in correlation with results previously published[[4](#_ENREF_4)] where carboxyl QDs induced a highly significant level of cell death following 3 cell cycles post cell exposure.

#### 3. Determination of free cellular cadmium ions

HFF-1 cells were seeded at 3 × 10^5^ cells/mL and placed in 75 cm^2^ cell culture flasks and allowed to settle overnight. The following day cells were treated with c_QD_ = 15 nM of amine (QD+) or carboxyl (QD-) QDs and incubated for 24 h. The treatment concentration was selected based on previous data where we detected significant levels of toxicity at this dose. Following exposure, treated cells were washed three times with sterile phosphate buffered saline (PBS) and fresh medium containing 60 µM Apigenin (Sigma–Aldrich, Bornem, Belgium), which halted cellular proliferation, were added. Cells were incubated during the period of the experiments. On days 1, 2, 3, 4, and 5 cells were detached, centrifuged at 0.4 rcf for 6 min, suspended in sterile PBS, and counted using a haemocytometer. For every measurement, 2 × 10^6^ cells were removed from the total number of cells. These cells were lysed with 50 μL dimethyl sulfoxide (DMSO) and transferred into black 96 well plates in 10 μL aliquots, which were supplemented with 200 μL per well of Measure-IT assay solution. Cadmium ion concentrations were determined at 490 nm excitation and 520 nm emission wavelength as per manufacturer's instructions, using the Cd^2+^ calibration curve provided with the assay kit. Measurements were taken on a Wallac Envision plate reader. Data are expressed as mean ± standard error of the mean (SEM) for three independent experiments.

**4. Evaluation of cellular excretion of QDs**

Cells were seeded in 25 cm² tissue culture flasks at 1x10^5^ cells/mL and allowed to attach overnight. Then, media were removed and fresh media were given containing the amine- or carboxyl-QDs at c_QD_ = 0, 2.5, 7.5 or 15 nM. Cells were incubated with the QDs for 4 or 24 h. Following incubation, media were removed, cells were washed 3x with PBS, and fresh media was given. The cells were then placed back in a humidified atmosphere at 37 °C and 5% CO_2_. After 30, 120, 240 or 360 min, 200 µL samples were taken per sample and stored at 4 °C prior to being processed for analysis with inductively coupled plasma-mass spectrometry. Five measurements were taken per sample. All samples were diluted due to the necessity to digest the QDs with Aqua Regia. Therefore all raw data must were multiplied with the used dilution factor.

**5. High content cellular image analysis**

High content (HC) imaging studies were conducted as described before.[[5](#_ENREF_5)] Briefly cells were seeded in 24 well plates (Nunc, Belgium) at 1.5x10^4^ cells/mL in 500 μL culture media and allowed overnight incubation in a humidified atmosphere at 37 °C and 5% CO_2_. The following day cells were exposed to the amine or carboxyl QDs for 4 h or 24 h in their full growth medium at c_QD_ = 0, 2.5, 5, 7.5, 10, and 15 nM concentration. Measurement probing for cytoskeletal effect, cellular and mitochondrial reactive oxygen species (ROS), and LC3 protein induction studies, were carried out.

**5.1 InCell analysis of cytoskeletal changes**

Following exposure to QDs, cells were washed twice with PBS. One set of 24 h exposure samples were immediately fixed with 4% paraformaldehyde (PFA). Another set of 4 h and 24 h exposure samples were allowed additional 24 h incubation. This was followed by the removal of the QDs before being fixed in 4% PFA, in order to account for a delayed effect of the QDs on the cellular cytoskeleton. Cells were then permeabilised with Triton X-100 (1%) and blocked with 10% serum-containing PBS, followed by room temperature incubation with primary anti-tubulin antibody (Abcam, Cambridge, UK). Cells were washed twice with PBS and incubated with secondary AF488-coupled secondary anti-tubulin antibody (Molecular Probes, Life Technologies Europe, BV, Belgium) and AF568-coupled phalloidin (Molecular Probes, Life Technologies Europe, BV, Belgium). Finally, cells were stained with CellMask blue (Molecular Probes, Life Technologies Europe, BV, Belgium) for cytoplasmic/nuclear visualisation. Afterwards cells were kept in PBS until ready for InCell analysis (In Cell analyser 2000, GE Healthcare Europe GmbH, Belgium). Investigator Tool 1.6.1 was used for segmenting cells and analysing fluorescence intensities in individual cells. Approximately 2000 cells were analysed per sample per replicate. Results were expressed as the mean + standard error to the mean of the untreated control.


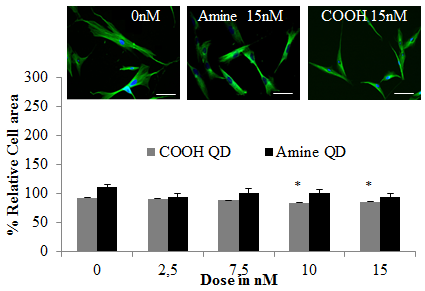


**Figure S12.** Cell morphology associated defects induced by amine- (QD+) and COOH- (QD) QDs in HFF-1 cells. Control cells and cells were exposed to the respective QDs up to doses of c_QD_ = 15 nM following high-content analysis of cells stained with CellMask (blue: cytoplasm), α-tubulin (green) and F-actin (red). Quantitative data are shown for analysis of a minimum of 2000 cells per condition per replicate for 24 h QD exposure at various concentrations. Quantitative data are presented as mean + SEM (*n* = 3) for QD-treated cells relative to untreated control cells (= 100%). The degree of statistical significance is shown when appropriate (*: p < 0.05, **: p < 0.01, ***: p < 0.001). The inset images are representative high-content images of cells stained with the respective QDs at 15 nM (scale bar = 100 µm).

**5.2 InCell ROS analysis**

For this part of the experiments following exposure to QDs for 4 h and 24 h at the above mentioned doses, QDs were removed by washing with PBS. Cells were stained with CellROX green (Molecular Probes, Life Technologies Europe, BV, Belgium) for nuclear, and cytoplasmic ROS detection, followed by staining with MitoTracker Red CMXRos (Molecular Probes, Life Technologies Europe, BV, Belgium) for mitochondrial ROS detection. Finally, cells were fixed with 4% PFA and preserved in the cold in PBS away from light. Plates were analysed on the InCell analyser 2000 as described above.


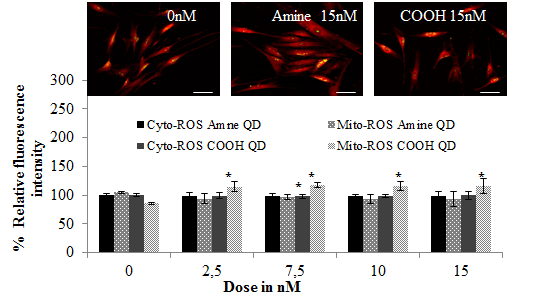


**Figure S13**. Induction of ROS by amine- and COOH-QDs in HFF-1 cells exposed to various concentrations for 24 h. Data are shown for high-content imaging based results for cells stained with CellROX Green (cytoplasmic ROS) and MitoTracker Red CMXRos (mitochondrial ROS). Quantitative data is shown for analysis of a minimum of 2000 cells per condition per replicate. Quantitative data are presented as mean + SEM (*n* = 3) for QD-treated cells relative to untreated control cells (= 100%). The degree of statistical significance is shown when appropriate (*: p < 0.05, **: p < 0.01, ***: p < 0.001). The inset images are representative high-content images of control cells and cells stained with the respective QDs at c_QD_ = 15 nM (scale bar = 100 μm).

**5.3 InCell LC3 analysis**

The expression of the LC3 protein was investigated in the HFF-1 cells treated with amine and carboxyl QDs for 4 h and 24 h. Cells were prepared as mentioned above and following exposure, cells were washed twice with PBS. One set of 24 h exposure samples were immediately fixed with 4% PFA. Another set of 4 h and 24 h exposure samples were allowed additional 24 h incubation. This was followed by the removal of the QDs before cells were fixed in 4% PFA, in order to account for a delayed effect of the QDs on inducing autophagy. Following fixing, cells were permeabilised with Triton X-100 (1%), and blocked with 10% serum-containing PBS. Cells were then incubated in the dark at room temperature with primary murine anti-LC3 antibody (Cell Signalling Technologies, Belgium), followed by counterstained of the nuclei with 4′,6-diamidino-2-phenylindole (DAPI; Molecular Probes, Invitrogen, Belgium). Finally, cells were washed twice with PBS, and plates were analysed with the InCell analyser 2000 as described above.


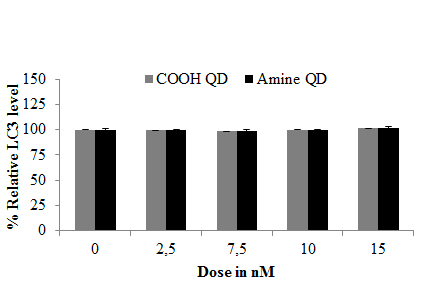


**Figure S14**. Cellular levels of the LC3 protein induced by amine- and COOH-QDs in HFF-1 cells. Control cells and cells were exposed to the respective QDs up to doses (in terms of QD concentration) of 15 nM, followed by high-content analysis of cells labelled with LC3-B antibody. Quantitative data are shown for analysis of a minimum of 2000 cells per condition per replicate for 24 h QD exposure at various QD concentrations. Quantitative data are presented as mean + SEM (*n* = 3) for QD-treated cells relative to untreated control cells (= 100%).

**6. Confocal microscopy of QD uptake and InCell analysis of changes to tubulin fibers**

Confocal microscopy with z-stacking was used to analyse the uptake of QDs. Cells were seeded at 4x10^4^ cells/well into Lab-Tek chamber slides (Thermo Scientific BVBA, Belgium) and allowed to settle. Cells were then incubated with amine or carboxyl QDs for 4 h or 24 h. Cells were then washed twice with PBS and fixed with 4% PFA. Fixed samples were then permeabilised with Triton X-100 (1%) and blocked with 10% serum-containing PBS, followed by room temperature incubation with primary anti-tubulin antibody (Abcam, Cambridge, UK). Cells were then washed twice with PBS and incubated with secondary AF488-coupled secondary anti-tubulin antibody (Molecular Probes, Life Technologies Europe, BV, Belgium) and AF568-coupled phalloidin (Molecular Probes, Life Technologies Europe, BV, Belgium). Prior to image acquisition, cells were stained with 1% Hoechst (Life Technologies Europe, BV, Belgium) nuclear counterstain. Fluorescence was captured with an inverted Nikon C1 confocal microscope (Nikon Corporation BVBA, Belgium). Each sample was acquired in 3D, whereby a series of images along the z-axis was obtained starting from the top of the cell towards the bottom. At least 20 slices were acquired per cell. The NIS-elements software was used for fluorescence acquisition and images were analysed using ImageJ software. At least 10 cells were analysed per QD type per condition. Results are expressed as the mean ± standard error.

**7. Endosomal / lysosomal and single particle tracking**

QD uptake *via* the endosomal and/or lysosomal route was tracked using the single particle tracking system (SPT system).

HFF-1 cells were seeded at 1.5 x 10^5^ cells/mL in 1 mL total culture media on sterile MatTek coverslips (1.5)-bottom dishes (MatTek Corporation, MA, USA). The next day 30 μL/mL Bac-Mam endosomal or lysosomal stains (Life Technologies Europe, BV, Belgium) were added to each plate in total culture medium. Each QD type was added to 1 mL OptiMEM to a final concentration of c_QD_ = 2.5 nM. Cells were imaged at various time points with time 0 equal to the time of addition of the QDs to the cells. Recordings and images were taken up to 6 h post treatment. In the experiments with the carboxyl-QDs time 0 represents 30 min following exposure, due to the delay with which these QDs to come into close proximity with the cell membrane.

Dual-colour imaging of live-cells for trajectory-based colocalization was conducted on a custom-built laser widefield fluorescence microscope (SPT). A TE2000-E inverted microscope was used with the SPT (Nikon Belux, Brussels, Belgium). Endosomal and lysosomal labels were excited with a 491 nm laser line (Cobolt, Stockholm, Sweden), which also corresponded with the excitation wavelength required for the QDs. Green and red fluorescence images were recorded simultaneously on separate halves of the chip with an electron-multiplying CCD (EMCCD) camera (Cascade II:512, Roper Scientific, Nieuwegein, The Netherlands). The videos were acquired in NIS Elements (Nikon Belux, Brussels, Belgium) mainly at a frame speed of 2 frames/second and exposure times of 100 ms. Quantitative colocalization analysis on the obtained results was performed using three methods respectively based on colocalization of pixels, objects, and object trajectories as described in elsewhere.[[6](#_ENREF_6)] Processed images were automatically thresholded and converted to binary images, which were used for calculating pixel overlap. Pixel overlap was based on the fraction of pixels from the red channel (QDs) that coincided with pixels from the green channel (endosomes or lysosomes). For object colocalization based analysis contours were drawn over automatically identified individual fluorescent objects (endosomes/lysosomes and QDs). A red object (ensemble of QDs) was assigned as being colocalized with a green object (endosome/lysosome), when the centroid of the red object was located inside the contour of the green object. Finally, the dynamic, trajectory-based colocalization was analysed using motion trajectories acquired *via* the recorded movies of the identified green and red objects. Algorithms in custom built MatLab software were utilised to perform calculations. The dynamic colocalization coefficient, which detected correlated movement between red and green objects, was thus the fraction of red trajectories that showed correlated movement with green trajectories.

**8. Colocalization of QDs with lysosomes or endosomes *via* confocal microscopy**

To assess the possible lysosomal or endosomal localization of the QDs, HFF-1 cells were seeded at 4x10^4^ cells/dish in collagen-coated 35 mm glass bottom MatTek dishes (MatTek Corporation, Ashland, MA, USA) in 1.5 mL of full culture medium. Cells were allowed to settle overnight prior to being incubated with either 60 nM of Lysotracker Green (Life Technologies Europe, BV, Belgium) for 1 hr at ambient temperature, or the lipophilic membrane tracer dye 3,3′-dioctadecyloxacarbocyanine perchlorate (DiO; Life Technologies, Europe, BV, Belgium) for 30 min at 2.5 µg/mL. Cells were then washed twice with PBS and incubated with 2.5 nM of amine or carboxyl QDs for 4 h at 37 °C in a humidified atmosphere. Subsequently, the media were removed, cells were washed three times with PBS and fixed with 4% PFA for 15 min at ambient temperature; prior to visualization using confocal microscopy (LSM 700 confocal laser-scanning microscope, Zeiss, Germany). For 3D image acquisition cells were exposed to DAPI nuclear counterstain for 10 min at ambient temperature, followed by two PBS washes and confocal visualization. For each cell, a series of images along the z-axis was obtained, starting from the top of the cell towards the bottom. At least 20 slices were acquired per cell.

Colocalization was analysed using the JACoP plugin in ImageJ. The degree of colocalization was calculated using thresholded Manders’ correlation coefficient of global statistical analysis, considering pixel intensity distributions. At least 10 z-stacks were analysed per sample, and the percentage of the fraction of each type of QDs in the endosomes or lysosomes was expressed as the mean ± standard error of the mean.

**9. Gene expression studies**

For gene expression studies, the following RT-PCR gene pathways array were used: the human cytoskeletal regulatory pathway (PAHZ-088Z, Qiagen Benelux BV, Netherlands), and the inflammatory cytokines and receptor pathway (PAHS-011Z, Qiagen Benelux BV, Netherlands).

Briefly, cells were seeded at 1.5x10^5^ cells/mL and allowed to settle overnight. Cells were either left untreated (negative controls) or incubated with c_QD_ = 2.5, 7.5, and 15 nM amine or carboxyl QD concentrations for 24 h. Next, cells were washed twice with PBS and harvested into centrifuge tubes. RNA was extracted using the Qiagen RNeasy Mini Kit (Qiagen, Benelux BV, Netherlands) according to the manufacturer’s instructions. Each RNA sample underwent DNase digestion to eliminate genomic DNA contamination, using the RNase-Free DNase Set (Qiagen, Benelux BV, Netherlands). RNA samples were converted into first strand cDNA using the RT^2^ First Strand Kit (Qiagen Benelux BV, Netherlands), whereby Genomic DNA Elimination Mixture was applied according to the manufacturer's instructions and samples were PCR amplified. RT^2^ qPCR Master Mix plus SybrGreen (Qiagen Benelux BV, Netherlands) was used to prepare the RT-PCR mixture, which was run on the iCycler iQ5 Thermal Cycler (Bio-Rad Laboratories N.V., Belgium). PCR array data was analysed using the ΔΔC_t_ method *via* the SABiosciences web portal ([www.SABiosciences.com/pcrarraydataanalysis.php](http://www.SABiosciences.com/pcrarraydataanalysis.php)).

**10. Supporting references**

1. Yu H, Li J, Loomis RA, Wang LW, Buhro WE: **Two- versus three-dimensional quantum confinement in indium phosphide wires and dots.** *Nat Mater* 2003, **2:**517-520.

2. Soenen SJ, Montenegro JM, Abdelmonem AM, Manshian BB, Doak SH, Parak WJ, De Smedt SC, Braeckmans K: **The effect of nanoparticle degradation on amphiphilic polymer-coated quantum dot toxicity: the importance of particle functionality assessment in toxicology [corrected].** *Acta Biomater* 2014, **10:**732-741.

3. Soenen SJ, Demeester J, De Smedt SC, Braeckmans K: **The cytotoxic effects of polymer-coated quantum dots and restrictions for live cell applications.** *Biomaterials* 2012, **33:**4882-4888.

4. Manshian BB, Soenen SJ, Al-Ali A, Brown A, Hondow N, Wills J, Jenkins GJ, Doak SH: **Cell Type-Dependent Changes in CdSe/ZnS Quantum Dot Uptake and Toxic Endpoints.** *Toxicol Sci* 2015, **144:**246-258.

5. Manshian BB, Moyano DF, Corthout N, Munck S, Himmelreich U, Rotello VM, Soenen SJ: **High-content imaging and gene expression analysis to study cell-nanomaterial interactions: the effect of surface hydrophobicity.** *Biomaterials* 2014, **35:**9941-9950.

6. Vercauteren D, Deschout H, Remaut K, Engbersen JF, Jones AT, Demeester J, De Smedt SC, Braeckmans K: **Dynamic colocalization microscopy to characterize intracellular trafficking of nanomedicines.** *ACS Nano* 2011, **5:**7874-7884.
